# Genomic analysis, culturing optimization, and characterization of *Escherichia* bacteriophage OSYSP, previously studied as effective pathogen control on fresh produce

**DOI:** 10.3389/fmicb.2024.1486333

**Published:** 2024-12-09

**Authors:** Mustafa Yesil, En Huang, Xu Yang, Ahmed E. Yousef

**Affiliations:** ^1^Department of Food Science and Technology, The Ohio State University, Columbus, OH, United States; ^2^Department of Environmental Health Sciences, University of Arkansas for Medical Sciences, Little Rock, AR, United States; ^3^Nutrition and Food Science Department, California State Polytechnic University, Pomona, CA, United States; ^4^Department of Microbiology, The Ohio State University, Columbus, OH, United States

**Keywords:** food safety, foodborne disease, biocontrol, *Escherichia coli* O157:H7, antibiotic resistance, whole genome sequencing, therapeutics

## Abstract

Advances in bacteriophage genome sequencing and regulatory approvals of some bacteriophages in various applications have renewed interest in these antibacterial viruses as a potential solution to persistent food safety challenges. Here, we analyzed in depth the genome of the previously studied *Escherichia* bacteriophage OSYSP (phage OSYSP), revealed its application-related characteristics, and optimized its enumeration techniques for facilitating industrial implementation. We previously sequenced phage OSYSP genome completely by combining results from Illumina Miseq and Ion Torrent sequencing platforms and completing the remaining sequence gaps using PCR. Based on the genomics analysis completed herein, phage OSYSP was confirmed as an obligate lytic phage of the *Caudoviricetes* class. The genome encodes 81 proteins of identifiable functions, including two endolysins and 45 proteins that support host-independent DNA replication, transcription, and repair. Despite its similarities to T5-like phages, unique genome arrangements confirm phage OSYSP’s novelty. The genomic analysis also confirmed the absence of DNA sequences encoding virulence or antibiotic resistance factors. For optimizing phage detection and quantification in the conventional plaque assay, it was observed that decreasing the concentration of agar or agarose, when used as a medium gelling agent, increased phage recovery (*p* < 0.05), but using agarose resulted in smaller plaque diameters (*p* < 0.05). Phage OSYSP inactivated pathogenic and non-pathogenic strains of *E. coli* and some *Salmonella enterica* serovars, with more pronounced effect against *E. coli* O157:H7. Phage titers remained fairly unchanged throughout a 24-month storage at 4°C. Incubation for 30 min at 4°C−47°C or pH 4–11 had no significant detrimental effect (*p* > 0.05) on phage infectivity. *In vitro* application of phage OSYSP against *E. coli* O157:H7 EDL933 decreased the pathogen’s viable population by >5.7-log CFU/mL within 80 min, at a multiplicity of infection as low as 0.01. The favorable genome characteristics, combined with improved enumeration methodology, and the proven infectivity stability, make phage OSYSP a promising biocontrol agent against pathogenic *E. coli* for food or therapeutic applications.

## 1 Introduction

The widespread use of antibiotics in human medicine, and in food animal production, accelerated the emergence of antimicrobial resistance among bacterial pathogens (Potera, 2013). Consequently, antibiotics that were once effective in controlling pathogenic bacteria are no longer effective, resulting in prolonged illness durations and increased mortality (World Health Organization, 2014). Moreover, antibiotic residues and antimicrobial-resistant bacteria have been detected in essential food groups, such as milk, eggs, meat, and fresh produce (Arsène et al., 2022; Friedman, 2015). The spread of antimicrobial resistance among foodborne pathogens amplifies disease hazards linked to these pathogens, particularly with the expected rise in food distribution and the emergence of new pathogenic bacteria (Center for Disease Control and Prevention, 2024). Use of bacteriophages (phages) in lieu of antibiotics (World Health Organization, 2024) may assist in suppressing the antimicrobial resistance trajectory that has been rising for decades.

Recently, food and medical microbiologists are paying great attention to bacteriophages for their potential application in eliminating bacterial pathogens. Bacteriophages are the most ubiquitous biological entities on Earth, with an estimated number of 10^31^ (Hendrix et al., 2002), and the most abundant viral particles in the human gastrointestinal tract (Lusiak-Szelachowska et al., 2017). Phages can even be found in human saliva and dental plaques (Bachrach et al., 2003; Hitch et al., 2004). *Escherichia coli*-specific phages have been isolated from various raw and processed food products, including fresh produce, chicken meat, pork, and ground beef (Kennedy et al., 1986). Application of bacteriophages, alone or in combination with other antimicrobial agents, was found effective in controlling pathogenic microorganisms on food (Snyder et al., 2016; Leverentz et al., 2003). As therapeutic agents, phages have eliminated pathogenic microorganisms and promoted livestock growth when administered via oral route (Miller et al., 2010) or incorporated into animal feed (Kim et al., 2014). Additionally, phages have been successfully explored as agricultural pesticides (Lim et al., 2013) and as sanitizers to reduce the pathogens on processing equipment (Tomat et al., 2014). Owing to their efficacy across various applications, numerous bacteriophage products were approved by regulatory agencies and have been commercially available in the United States. Notable among these are Salmonelex by Micreos for treating raw meat and poultry, ListShield, EcoShield, and SalmoFresh by Intralytix for use in food and on food contact surfaces, and AgriPhage by OmniLytics as an agricultural pesticide for treating tomato and pepper infections (Sulakvelidze, 2013).

Despite the progress made in phage research and applications, it is important to characterize new effective phages against the current and emerging pathogens, particularly those acquiring antibiotic resistance. Such novel phages need to be fully characterized to ensure their suitability for food and clinical applications. Genetic characterization of new phages reveals antimicrobial resistance or toxin encoding genes, if present, identify their replication cycles, and detects potential allergens within their genomes. Similarly, improving culture-based characterization can assist in proving phages potency, and determining their purity and stability under challenging environmental conditions.

The anti-*E. coli* bacteriophage OSYSP (phage OSYSP), used in current study, was originally isolated in this laboratory (Snyder et al., 2016). This phage was potent against *E. coli* O157:H7, whether used alone or coupled with gaseous ozone, for fresh produce decontamination (Snyder et al., 2016; Yesil et al., 2023). The goal of the current study was to fill the gaps in knowledge that is essential for the usability of phage OSYSP as a new pathogen control agent. This goal can be realized by implementing the following objectives: (a) characterizing phage OSYSP by thoroughly analyzing its genome, (b) improving phage’s culture-based detection and enumeration technique, and (c) determining the stability and longevity of the phage under different storage conditions. The analysis results should be beneficial in improving the feasibility of using phage OSYSP, and similar phages, for food safety or therapeutic applications.

## 2 Materials and methods

### 2.1 Bacterial strains, growth media, and enumeration conditions

*Escherichia coli* O157:H7 EDL933 and *E. coli* O157:H7 B6-914 were used for phage OSYSP’s propagation and enumeration, respectively. *E. coli* and *Salmonella enterica* strains were cultured using Luria-Bertani (LB) growth medium (Becton Dickinson, Sparks, MD, USA). To prepare a fresh bacterial culture, a single colony grown on a suitable agar medium, was transferred into fresh LB broth and incubated at 37°C for 15 h; this was followed by a second transfer under the same conditions. The resulting culture was centrifuged at 5,500 × *g* for 10 min in a benchtop centrifuge (Model Centra MP4R; International Equipment Co., Nashville, TN, USA). The culture supernatant was then removed, and the remaining cell pellet was suspended in 0.1% (wt/vol) buffered peptone water (BPW; Becton Dickinson) for use in further testing. For bacterial enumeration, serial dilutions of cultures or cell suspensions were prepared in BPW and spread-plated on LB agar plates, which were then incubated at 37°C for 24 h. Unlike the other strains, a modification was made to the growth medium for *E. coli* O157:H7 B6-914. This strain contains genes encoding ampicillin resistance and green fluorescence proteins for easy enumeration. Therefore, the growth and enumeration media for *E. coli* O157:H7 B6-914 were supplemented with 100 μg/mL ampicillin (Fisher Scientific, Fairlawn, NJ, USA), whereas all other cultivation conditions remained the same.

### 2.2 Phage OSYSP propagation and purification

Phage OSYSP was previously isolated from municipal wastewater (Snyder et al., 2016). In the current study, a systematic approach was developed for the optimal phage cultivation conditions. To propagate the phage OSYSP, 30-μL pure phage stock (10^9^ PFU/mL) were mixed with 3 mL *E. coli* O157:H7 EDL933 overnight culture (10^9^ CFU/mL) at a multiplicity of infection (MOI) of 0.01. The phage-host mixture was dispensed in 30-mL LB broth supplemented with 2 mM CaCl_2_ (Fisher Chemical) and incubated at 37°C for up to 3 h. The propagated phage mixture was centrifuged at 5,500 × *g* for 10 min and bacterial cells were removed by microfiltration using a 0.45 μm filter unit (Merck Millipore Ltd., Cork, Ireland). The filtrate served as a host-free phage lysate. Thereafter, ultracentrifugation was performed to replace the LB broth base and remove other impurities from phage lysate. Briefly, phage lysates were centrifuged at 58,000 × *g* for 3 h using an ultra-speed centrifuge (Beckman L8-55; Beckman Instruments co., Palo Alto, CA, USA) equipped with a suitable rotor (Ti 70 rotor; Beckman Instruments co.). After the supernatant was removed, the undisturbed phage pellet was re-suspended in BPW to obtain the pure phage suspension.

### 2.3 Modified double-layer plaque assay for phage titer determinations

A modified double-layer plaque assay was used throughout this study to quantify phage OSYSP. The impact of the type and concentration of gelling agents used in the soft overlay, against phage titer and plaque diameter, was investigated. The double-layer plaque assay consists of a top (soft) and a bottom (solid) layer. To prepare the bottom layer of the growth medium, LB agar plates were prepared according to the manufacturer’s instructions by adding 1.5% agar (Fisher Scientific) into the LB broth medium before sterilization. To test the influence of gelling agent type (in the top layer) and concentration against phage titer and plaque diameter, the double-layer plaque assay was performed as follows. A final concentration of 0.2, 0.3, 0.5 or 0.75% agar or agarose (Fisher Scientific), as gelling agent, was added into the LB broth (top layer) during medium preparation before sterilization. Serial dilutions of pure phage suspension were prepared in BPW, and 100 μL were combined with 200 μL *E. coli* O157:H7 B6-914 cell suspension. The phage-host preparation was mixed in a 10 mL LB top overlay medium at 47°C and poured on LB agar plates supplemented with 100 μg/mL ampicillin. Plates were kept at 20°C for 30 min to allow the overlay to harden. After overnight incubation at 37°C, clear phage plaques on agar plates were counted to determine phage titers as plaque forming units (PFU)/mL. To measure phage plaque diameters, Petri plates with phage plaques were photographed using a gel documentation system (Universal Hood II; Bio-Rad, Hercules, CA, USA) with a fluorescent ruler for scale. Image analysis program (ImageJ; National Institute of Health) was used to measure the phage plaque diameters from the obtained images (Abràmoff et al., 2004).

### 2.4 One-step growth curve of phage OSYSP

Phage OSYSP replication curve was developed to determine the phage proliferation dynamics and to observe its lytic life cycle. In this experiment, 30-μL pure phage suspension (10^9^ PFU/mL) and 3 mL overnight culture of the host, *E. coli* O157:H7 EDL933 (10^9^ CFU/mL), were mixed, producing a multiplicity of infection of 0.01. The mixture was incubated at 37°C for 20 min to allow host infection with the phage. Following incubation, the mixture was centrifuged at 15,500 × *g* in a benchtop centrifuge (International Equipment Co.) for 10 min to remove free phages from the medium. The remaining phage-infected cells (pellet) were suspended in 30 mL LB broth supplemented with 2 mM CaCl_2_; this represented time zero of the propagation. The suspension was incubated at 37°C for up to 2 h. At 0, 10, 20, 25, 30, 40, 70, 80, 100, and 120 min of propagation, 2-mL samples were collected from the propagation medium. At each sampling time point, host cells were removed by centrifugation at 14,900 × *g* for 2 min in a bench-top centrifuge (Biofuge; Heraeus Seperation technik Gmbh, Germany). The phage titer was determined from the phage-containing supernatant by modified double-layer plaque assay, as described earlier.

### 2.5 *In vitro* potency of phage OSYSP against *Escherichia coli* O157:H7 EDL933

An experiment, similar to that reported in section 2.4, was conducted to determine the populations of bacterial hosts in the presence of phage OSYSP during 80 min of propagation. *E. coli* O157:H7 EDL933 and phage OSYSP populations were counted, as described earlier, before and after a 20-min pre-infection period, and also at the end of the propagation period. Additionally, the optical density (OD; *λ* = 600 nm) of the propagation medium was measured using a spectrophotometer (Spectronic Genesys 5; Milton Roy Co., Rochester, NY, USA) at 0, 10, 20, 25, 30, 40, 50, 60, 70, and 80 min of incubation to construct the corresponding OD-based survival curve.

### 2.6 Host range determination

The host range of phage OSYSP was determined by the spot test and the double-layer plaque assay, covering 5 *E. coli* and 12 *S. enterica* strains. The analysis included confirmation of phage OSYSP activity against strains reported previously by Snyder et al. (2016). To test the host range with spotting method, 10 μL of phage suspension (10^9^ PFU/mL stock, and its decimal dilutions) or phage-free BPW (control) was spot-inoculated onto LB agar plates seeded with the target bacteria. Phage-spotted agar plates were incubated overnight at 37°C. To test the host range with double-layer plaque assay, phage stock (10^9^ PFU/mL) was serially diluted with BPW and mixed with the target bacteria (strains of *S. enterica* and *E. coli*) in an LB top overlay at 47°C. This mixture was then poured on Petri plates containing LB agar base layer, as previously described, for phage enumeration.

### 2.7 Stability of phage OSYSP during extended refrigerated storage

Pure phage OSYSP suspension was prepared in phosphate buffered saline (PBS, pH 7.4), following the propagation and purification methods previously described. This phage stock was transferred to a sterile 15-mL glass test tube tightly covered with aluminum foil. The test tube was stored at 4°C throughout the 24-month sampling period. Phage titers were periodically determined using double-layer plaque assay.

### 2.8 pH sensitivity assay

LB broth media (pH 6.9) were prepared following manufacturer’s instructions and adjusted to the final pH values ranging from 2 to 12, using sodium hydroxide (Jenneile Enterprises., Cincinnati, OH, USA) or hydrochloric acid (Fisher Chemical). The media with modified pH were then filter sterilized using 0.45 μm syringe-driven filter units. To test phage sensitivity to pH, 300-μL pure phage suspensions (9.6 log PFU/mL) were mixed with 2,700-μL sterile pH-modified LB broths. The pH value of the incubation media was confirmed after the addition of the phage suspension. The mixtures of phages and media at various pH values were left at room temperature (23°C) for 30 min to allow interaction. Phage titers were determined after the treatment using the double-layer plaque assay.

### 2.9 Survival of phage OSYSP held at different incubation temperatures

To test the stability of phage OSYSP held at various temperatures, a previous procedure for heat treating phage OSYSP (Yesil et al., 2024) was followed. Thus, 100 μL of phage suspension in BPW (9.7 log PFU/mL) was transferred into sterile PCR tubes, and heat-treated at 4, 25, 37 or 47°C for 30 min using a thermal cycler (GeneAmp PCR System 2400; Perkin Elmer, Norwalk, CT, USA). After each treatment, heated-suspension was immediately transferred into an ice-water bath for 5 min to normalize the temperatures. Titers of treated phage were determined by double-layer plaque assay.

### 2.10 Bacteriophage DNA extraction and whole genome sequencing

Bacteriophage genomic DNA was extracted from pure phage OSYSP stock using Norgen Phage DNA extraction kit (Norgen Biotek Corp., Ontario, Canada), according to the manufacturer’s instructions. The whole genome of phage OSYSP was sequenced twice both using Illumina Miseq sequencing platform at Penn State University (University Park, PA, USA) and Ion Torrent sequencing platform at the Ohio State University (Columbus, OH, USA). Procedure details and brief findings were reported previously (Yesil et al., 2017).

### 2.11 Phage OSYSP genome assembly and annotations

*De novo* assembly of paired end reads (2 × 250 bp) from Illumina Miseq platform was accomplished using SPAdes v. 3.10.1 genome assembler (Bankevich et al., 2012). Progressive Mauve algorithm (Rissman et al., 2009) was used to align and re-order the outcome (contigs) of SPAdes assembler with other relevant phages. Reads from Ion Torrent next-generation sequencing platform were used to confirm the arrangement of phage OSYSP genome, along with Sanger sequencing of PCR amplicons, as briefly described in a previous publication (Yesil et al., 2017) and detailed herein. Primers designed for gap filling and assembly confirmation of the genome sequence of phage OSYSP are shown in Table 1. To complete the genome of phage OSYSP, primer sets were designed, and the regions of interest were amplified using a ready-to-use polymerase solution (MyTag Red Mix 2x; Bioline, Boston, MA, USA) at the following PCR conditions: 50 μL reaction mixture was exposed to an initial denaturation temperature in a thermal cycler (Bio-Rad MJ Mini; Bio-Rad) at 95°C for 1 min followed by 35 cycles of 95°C for 15 s, each, 58°C for 15 s and 72°C for 10 s. PCR process was completed with an extension step at 72°C for 10 min and cooling the PCR products to 4°C. Two additional sets of primers were designed to rule out the phage genome misassembly and confirm the genome arrangement. PCR reactions were performed at the same conditions as just described with a modification to the annealing temperature from 58 to 63°C.

**Table 1:** Sets of PCR primers used in this study.

	^*^Primers	Nucleotide sequence (5′–3′)	Expected product size (bp)
Enterohemorrhagic *Escherichia coli* O157:H7 confirmation	*Stx*_1_ Forward	ACACTGGATGATCTCAGTGG	614
	*Stx_1_* Reverse	CTGAATCCCCCTCCATTATG	
	*Stx_2_* Forward	CCATGACAACGGACAGCAGTT	779
	*Stx_2_* Reverse	CCTGTCAACTGAGCAGCACTTTG	
	*eaeA* Forward	GTGGCGAATACTGGCGAGACT	890
	eae*A* Reverse	CCCCATTCTTTTTCACCGTCG	
	*uidA* Forward	GCGAAAACTGTGGAATTGGG	252
	*uidA* Reverse	TGATGCTCCATCACTTCCTG	
Phage genome assembly gap filling	Forward	GGATATACATCTTATTATCCCA	733
	Reverse	GTTTCCATCAGTCAGGTATTCC	
Phage genome assembly confirmation	1 Forward	GATTACGTACGCGTGGGTGT	412
	1 Reverse	TCCGAACGCTGATTTCACGA	
	2 Forward	AGTTCGAACGAGAGTGGTCG	533
	2 Reverse	TCCGAACGCTGATTTCACGA	

* Primer pairs used for detecting Enterohemorrhagic *E. coli* genes were previously published for *stx1* and *stx2* (Gannon et al., 1992), *eaeA* (Gannon et al., 1997), and *uid*A (Cebula et al., 1995).

Phage OSYSP coding sequences and genes were predicted using the software Glimmer3 (Delcher et al., 2007) and GeneMarkS (Besemer et al., 2001). Phage OSYSP genes and protein products were searched in the non-redundant protein database of the National Center for Biotechnology Information (NCBI)- Basic Local Alignment Search Tool for Proteins (BLASTP) (Altschul et al., 1997). Annotation of the complete genome of phage OSYSP was manually curated from a feature table, template, and FASTA files that were inputs to generate an output file (.sqn) on Sequin software from NCBI. The finalized and annotated complete genome sequence of phage OSYSP has been deposited in the NCBI GenBank under the accession number MF402939. In addition, PCR was carried out to detect genes encoding Shiga-toxins and other virulence factors (Table 1).

### 2.12 Genetic relatedness of phage OSYSP

To determine the genetic relatedness of phage OSYSP with similar phage genomes, average nucleotide identity (ANI) estimation was carried out by using JSpeciesWS online program (Richter et al., 2016). A phylogenetic tree of the related phages was constructed from the amino acid sequences of large terminase subunits or DNA polymerase using MEGA 7 software (Kumar et al., 2016). Briefly, the MUSCLE alignment algorithm tool (Edgar, 2004) was used to align the amino acid sequences of phage OSYSP with those of other phages known for their DNA packing mechanisms. The aligned output sequence files were then used to generate phylogenetic trees with the neighbor joining method in MEGA 7 software.

### 2.13 Statistical analysis

Each experiment was repeated at least three times using two replicates each. Bacterial and phage populations (CFU/mL and PFU/mL, respectively) were converted to logarithmic values before being subjected to the statistical analyses. Statistical analyses were performed using analysis of variance through the General Linear Model (GLM) procedure of Statistical Analysis System (SAS) v.9.3 (SAS Institute Inc., Cary, NC, USA). The independent variables included were propagation time, incubation temperature, pH, and the type and concentration of gelling agent, whereas the dependent variable was bacteria population, phage population, or phage plaque diameter. The analysis included mean comparisons using the least square mean (LSM) function in SAS, with the pair-wise difference option used to determine *p*-values. Differences were considered significant at *p* < 0.05.

## 3 Results

### 3.1 Genome based characteristics of phage OSYSP

**Figure 1:**
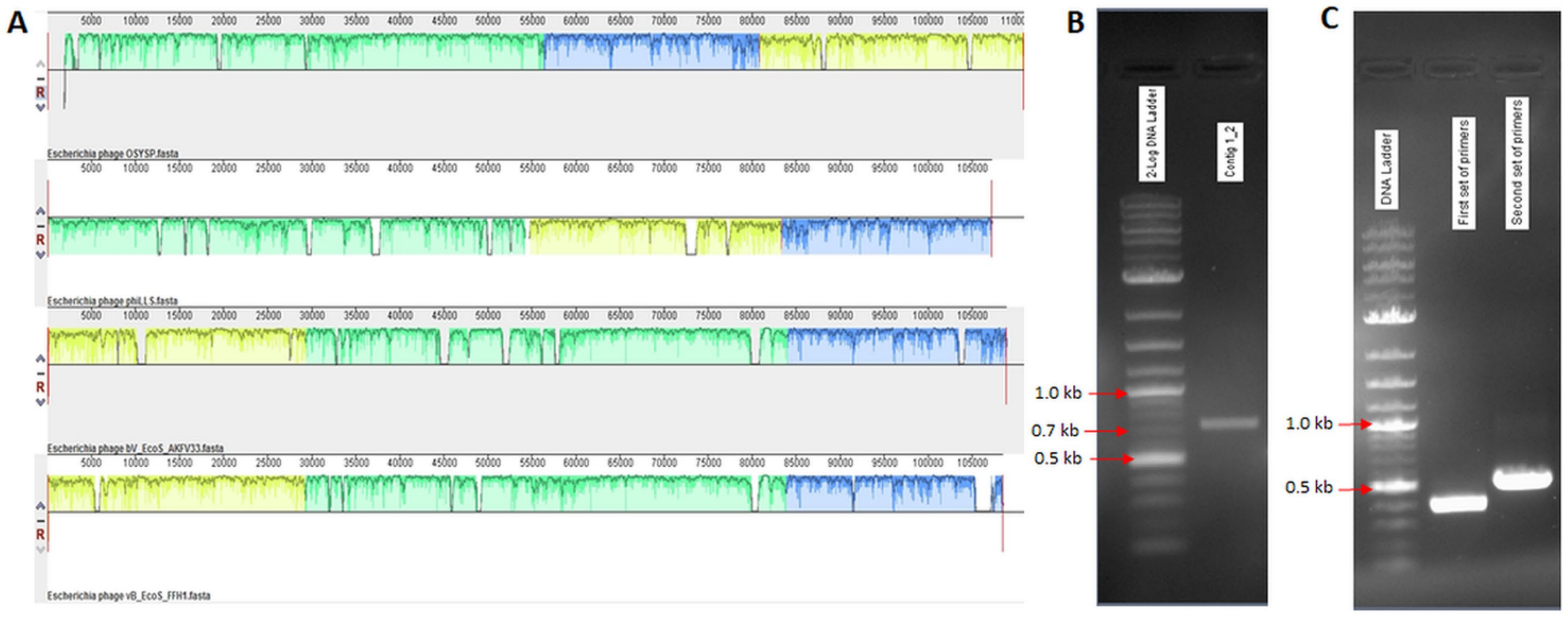
Alignment of *Escherichia* bacteriophage OSYSP genome with those of related phages. **(A)** Components from top to bottom are: OSYSP (MF402939.1); phiLLS (KY677846.1); AKFV33 (HQ6650.11); and FFH1 (KJ190157). **(B,C)** PCR products on agarose gels corresponding to primer sets in Table 1 for gap filling and genome assembly confirmation, respectively.

Two large contigs resulted from the *de novo* assembly of the Illumina Miseq raw data using SPAdes (v. 3.10.1) genome assembler software. These contigs were compared against the NCBI database, and reference complete genome sequences were obtained. Mauve alignment software was employed to align the phage OSYSP genome with related phage genomes, as shown in Figure 1A. Primer sets designed to verify genome assembly are presented in Table 1 with the expected sizes of the PCR product confirmed on agarose gel (Table 1; Figure 1B). PCR amplification followed by Sanger sequencing were used to rule out the possibility of a false genome assembly for the phage OSYSP (Figure 1C). Moreover, assembled reads from the Ion Torrent sequencing platform were used for further confirmation of PCR products and the phage genome arrangement.

The complete genome of phage OSYSP consists of a double-stranded DNA of 110,901 bp, with a GC content of 39.2%. *In silico* hybridization revealed that the complete genome of phage OSYSP shares high average nucleotide identities (ANIb) with several lytic phages. Specifically, phage OSYSP has ANIb values of 93.89% with T5-like poul124 (NC_047835.1), 93.12% with *Escherichia* phage APCEc03 (NC_047754.1), 93.11% with *Escherichia* phage phiLLS (KY677846.1), 93.10% with *Escherichia* phage vB_EcoS_FFH1 (NC_024139.1), 92.50% with *Escherichia* phage AKFV33 (HQ6650.11), and 90.59% with *Escherichia* phage T5 (NC_005859.1), as determined by JSpecies Web Server. A comprehensive comparison of the average nucleotide identity, genome size, GC content, and gene count between phage OSYSP and its closely related phages is shown in Table 2. Notably, the GC content of the phage OSYSP genome (39.2%) closely matches that of *Escherichia* phage T5 (NC_005859.1; 39.3%), but is considerably lower than its host strain, *E. coli* O157:H7 EDL933, which has an approximate G + C content of 50% (Kulasekara et al., 2009).

**Table 2:** *Escherichia* bacteriophage OSYSP average nucleotide identity and similarity comparison with closely related phages.

Bacteriophage	NCBI accession	Genome size (bp)	GC content (%)	ANIb (%)	Number of genes
*Escherichia* phage OSYSP	NC_047835.1	110,901	39.2	Reference genome	166
Bacteriophage T5-like poul124	MF431735.1	120,629	39.3	93.89	165
*Escherichia* phage APCEc03	NC_047754.1	103,737	38.9	93.12	151
*Escherichia* phage phiLLS	NC_047822.1	107,263	39.0	93.11	160
*Escherichia* phage vB_EcoS_FFH1	NC_024139.1	108,483	39.2	93.10	156
*Escherichia* phage vB_EcoS_AKFV33	NC_017969.1	108,853	38.9	92.55	160
*Escherichia* phage IrisVonRoten	MZ501075.1	112,239	39.0	92.53	172
*Salmonella* phage Spc35	NC_015269.1	118,351	39.4	91.73	145
*Salmonella* phage NR01	NC_031042.1	111,325	38.8	91.20	148
Phage NBSal005	NC_048857.1	121,040	39.1	90.92	178
*Escherichia* phage T5	NC_005859.1	121,750	39.3	90.59	162
*Escherichia* phage DT57C	NC_027356.1	108,065	39.7	90.31	133
Enterobacteria phage DT571/2	NC_047749.1	108,418	39.7	90.14	133
*Salmonella* phage Shivani	NC_028754.1	120,098	38.8	89.87	155
*Salmonella* phage Stitch	NC_027297.1	123,475	40.3	84.64	165
Enterobacteria phage EPS7	NC_010583.1	111,382	39.9	84.54	171

**Figure 2:**
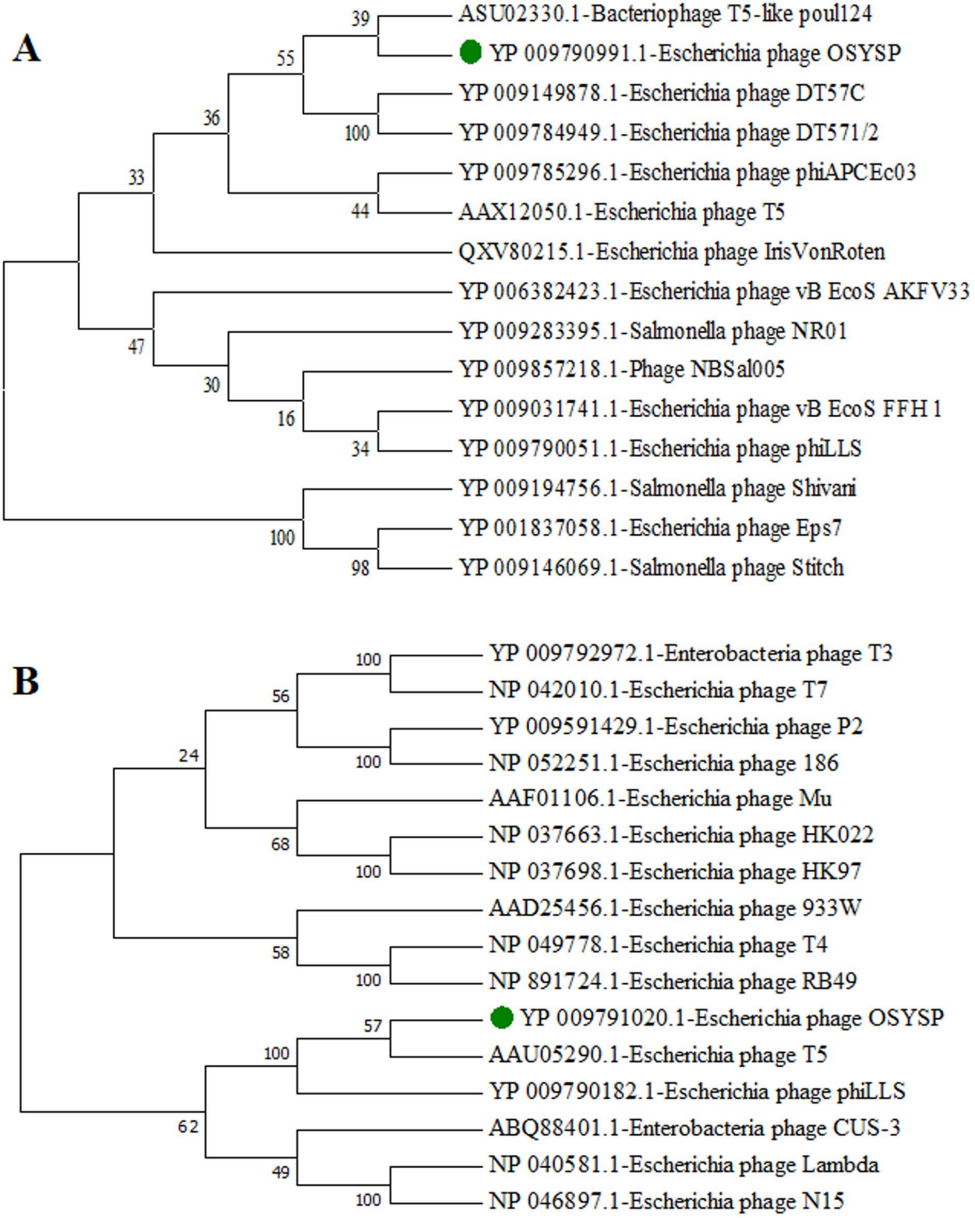
Phylogenetic trees for *Escherichia* bacteriophage OSYSP and related phages. The trees were constructed by aligning the amino acid sequences representing DNA polymerase from related bacteriophages **(A)**, and terminase large subunits from bacteriophages known for their DNA packaging mechanisms **(B)**. Phylogenetic trees were constructed using the neighbor-joining method, with 1,000 bootstrap replicates, and employing the Poisson model for genetic distance calculations in MEGA7 software.

To classify phage OSYSP further, phylogenetic trees were generated using the amino acid sequences from the DNA polymerases of related bacteriophages and terminase large subunits from the phages with well-known DNA packing mechanism (Figure 2). Phage OSYSP showed close genetic relatedness with other T5-like lytic phages. It clustered within the same clade and node as bacteriophage T5-like poul124 (NC_047835.1) and *Escherichia* phage T5 (NC_005859.1). Morphology of phage OSYSP revealed by transmission electron microscopy showed an isometric head with average dimensions of 98.6 nm in diameter and 105.7 nm in length, and a contractile tail measuring approximately 118.4 nm in length (Snyder et al., 2016). The Mauve alignment of phage OSYSP revealed significant similarities in homologous regions with T5-like phages such as *Escherichia* phage phiLLS (KY677846.1), *Escherichia* phage vB_EcoS_FFH1 (NC_024139.1), and *Escherichia* phage AKFV33 (HQ6650.11). However, notable genome rearrangements were identified among these phages. Taking into account the average nucleotide identity comparisons, phylogenetic tree constructions, transmission electron microscopy images, and Mauve analysis results, it can be asserted that phage OSYSP belongs to the *Caudoviricetes* class.

**Figure 3:**
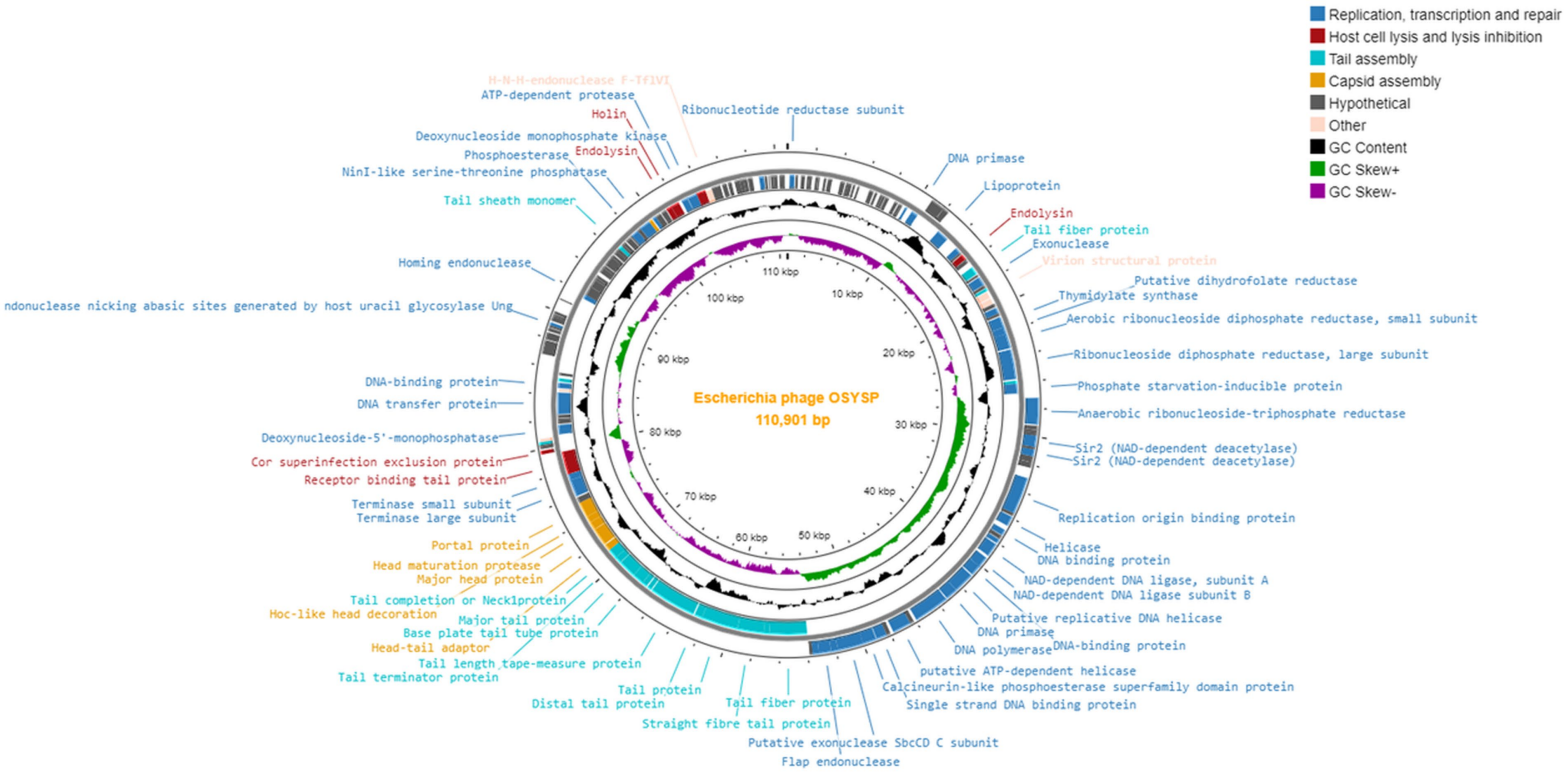
Genome map of *Escherichia* bacteriophage OSYSP.

Phage OSYSP genes were predicted by GeneMarkS, and their protein functions were obtained from the NCBI BLASTP database (Supplementary Table 1). The genome of phage OSYSP comprises 166 genes encoding eighty-one proteins with identifiable functions. Of these functional genes, 24 are located on the leading strand and 57 on the complementary strand. The functional proteins of phage OSYSP are involved in DNA replication, transcription and repair, host cell lysis and lysis inhibition, tail assembly, capsid assembly, and DNA packaging. A genome map generated using the Proksee web server^1^ detailing all proteins encoded by phage OSYSP is shown in Figure 3. Analysis based on the amino acid sequences revealed that phage OSYSP encodes proteins related to host-cell lysis. These include one holin (orf 147), two endolysins (orf 30 and orf 146), and *Rz*-like spanins (orf 150 and orf 151). The endolysins encoded in orf 30 (YP_009790953.1) and orf 146 (YP_009791069.1) are 100% identical to those found in *Salmonella* phage Spc35 (YP_004306556.1) and *Escherichia* virus AKFV33 (YP_006382340.1), respectively. The holin gene (YP_009791070.1) shares 99.54 and 99.08% similarities with *Escherichia* phage vB_Eco_mar004NP (YP_009824604.1) and *Salmonella* phage vB_Sen_I1 (QJA17897), respectively. Additionally, the two-component *Rz*-like spanins, encoded in orf 150 and orf 151, have similarities of 100 and 97.28% to the *Rz*-like spanins of *Escherichia* phage Eps7 (YP_001836970.1) and *Salmonella* phage S124 (YP_009806167.1), respectively.

Within phage OSYSP genome, there are 24 genes that encode proteins related to tail and capsid assembly. The proteins associated with capsid assembly, including major head proteins, head maturation protease, and Hoc-like head decoration proteins, were predicted to share similarities ranging from 98.77 to 100% with *Escherichia* and *Salmonella* phages. Phage OSYSP genome includes genes encoding tail structural proteins; these are the major tail protein, baseplate tail protein, tail fiber proteins, receptor binding tail proteins, and baseplate wedge protein. These genes have high sequence identities when compared to those in well-known lytic phages such as *Escherichia* phage phiLLS (KY677846.1), *Salmonella* phage Spc35 (NC_015269.1), and *Escherichia* phage vB_EcoS_FFH1 (NC_024139.1).

Phage OSYSP genome includes genes encoding functional proteins for packaging the phage inside its capsid; these include the terminase large and small subunits as well as a portal protein. Genes for these three proteins are found in orf 95, orf 97, and orf 98, respectively. These proteins exhibit similarities of 100, 99.77, and 99.38% to those from *Escherichia* phage vB_EcoS_FFH1 (YP_009031770.1), *Escherichia* phage T5 (YP_006983.1), and *Salmonella* phage Spc35 (YP_004306625.1), respectively.

The phage OSYSP genome encodes 45 functional proteins dedicated to phage DNA replication, transcription, and repair. Key proteins associated with nucleotide metabolism within the phage genome include ribonucleotide reductase subunit, ribonuclease H, helicases, DNA polymerase, DNA replication primase, thymidylate synthase, as well as small and large subunits of aerobic ribonucleoside diphosphate reductase. These proteins show high sequence similarity with those found in other phages: 100% with *Salmonella* phage Stitch (YP_009145996.1), 100% with *Escherichia* phage T5 (YP_006919.1), 100% with *Escherichia* phage DT57C (YP_009149867.1), 99.42% with *Salmonella* phage VSe12 (YP_009849652.1), 99.66% with *Escherichia* phage T5 (YP_006949.1), 100% with *Escherichia* phage T5 (YP_006920.1), 99.47% with *Escherichia* phage chee24 (YP_009795093.1), and 99.74% with *Escherichia* phage T5 (YP_006924.1). Furthermore, genes dedicated to transcription and repair, such as the putative transcriptional regulator and DNA binding proteins, show similarities to *Salmonella* phage Spc35 (YP_004306560.1, YP_004306587.1), *Escherichia* phage T5 (YP_006941.1), and *Escherichia* phage phiLLS (YP_009790170.1).

The phage OSYSP genome encodes 27 tRNA genes (Supplementary Table 2). It is essential for phage genomes not to encode genes linked to lysogeny, toxin production (such as Shiga toxin), or allergen proteins. Consistent with this requirement, none of these features were identified within the phage OSYSP genome. Additional PCR tests were conducted to experimentally verify the absence of Shiga toxin-encoding genes in the phage OSYSP genome. Phages encoding Shiga toxins can lead to the spread of these toxin genes, potentially resulting in the emergence of new Shiga toxin-producing *E. coli* strains (Herold et al., 2004).

**Figure 4:**
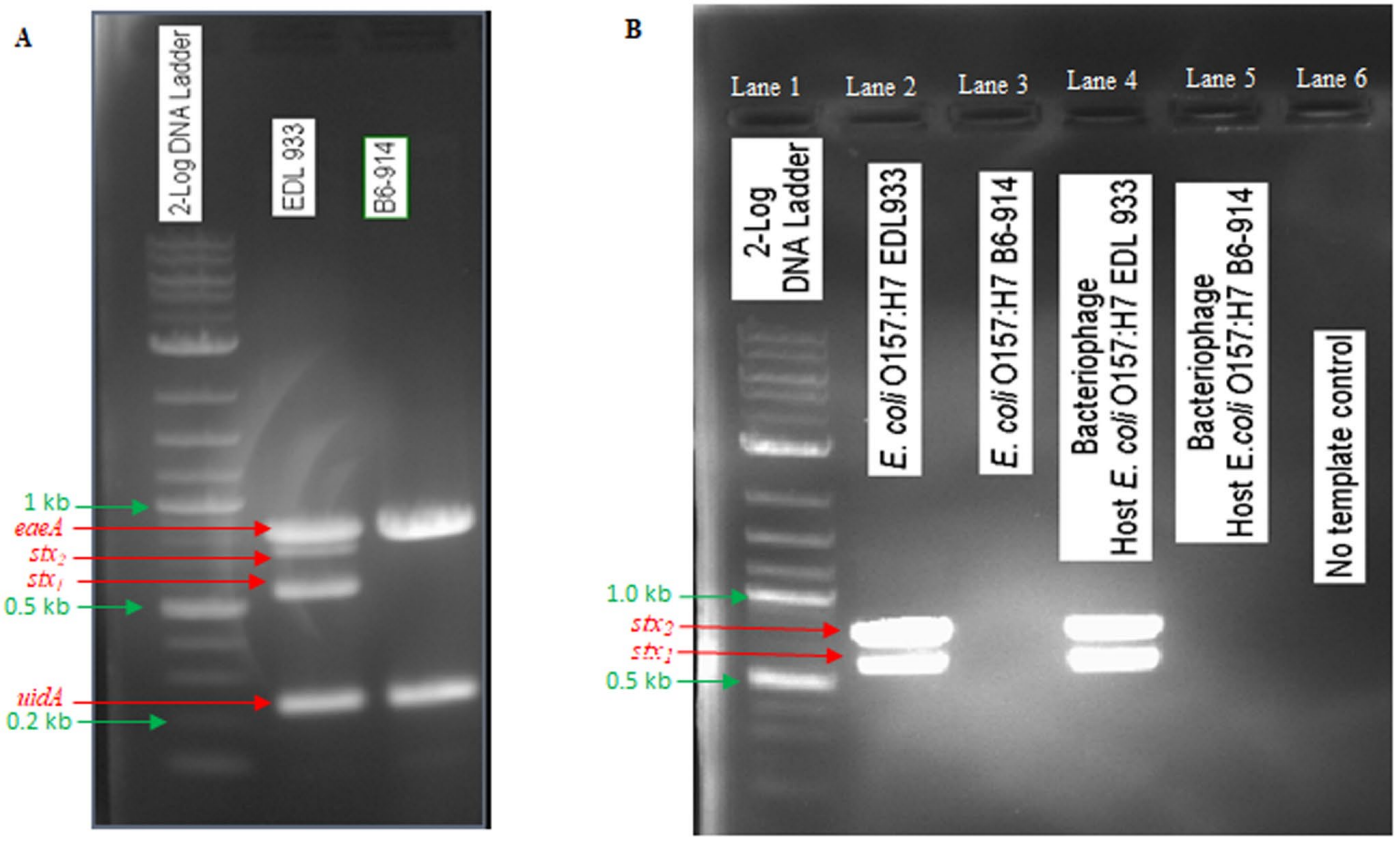
Detection of PCR gene products of Shiga toxins (*stxl, stx2*), intimin (*eaeA*), and β glucuronidase (*uidA*) in *Escherichia coli* O157:H7 EDL933 and *E. coli* O157:H7 B6-914, which were used in phage propagation and enumeration, and in *Escherichia* bacteriophage OSYSP (phage OSYSP) grown in these two host strains. **(A)** Host strains. **(B)** Host strains and phage OSYSP.

In this study, *E. coli* O157:H7 strain EDL933 was used for phage OSYSP propagation, while *E. coli* O157:H7 strain B6-914 served for its enumeration. *E. coli* O157:H7 EDL933 possesses genes encoding intimin, *β*-glucuronidase, Shiga toxin 1, and Shiga toxin 2. Conversely, *E. coli* O157:H7 B6-914 is a mutant strain devoid of Shiga toxin-encoding genes (Figure 4A). When propagating phage OSYSP using the Shiga toxin-producing *E. coli* O157:H7 EDL933, there was evidence on the electrophoretic gel for the presence of Shiga toxins in phage DNA extract (Figure 4B; Lane 4), despite the absence of such genes in phage genome, as indicated earlier. In contrast, using the *stx*-knockout strain, *E. coli* O157:H7 B6-914 (Figure 4B; Lane 5), for bacteriophage propagation did not show any *stx* bands on the gel. It is presumed that the *stx* bands in lane 4 represent a false positive outcome, most likely due to contamination of extracted phage DNA with that of the host bacterium (i.e., *E. coli* O157:H7 EDL933).

### 3.2 Improving phage OSYSP capturability by modifying gelling agent of the recovery medium

**Figure 5:**
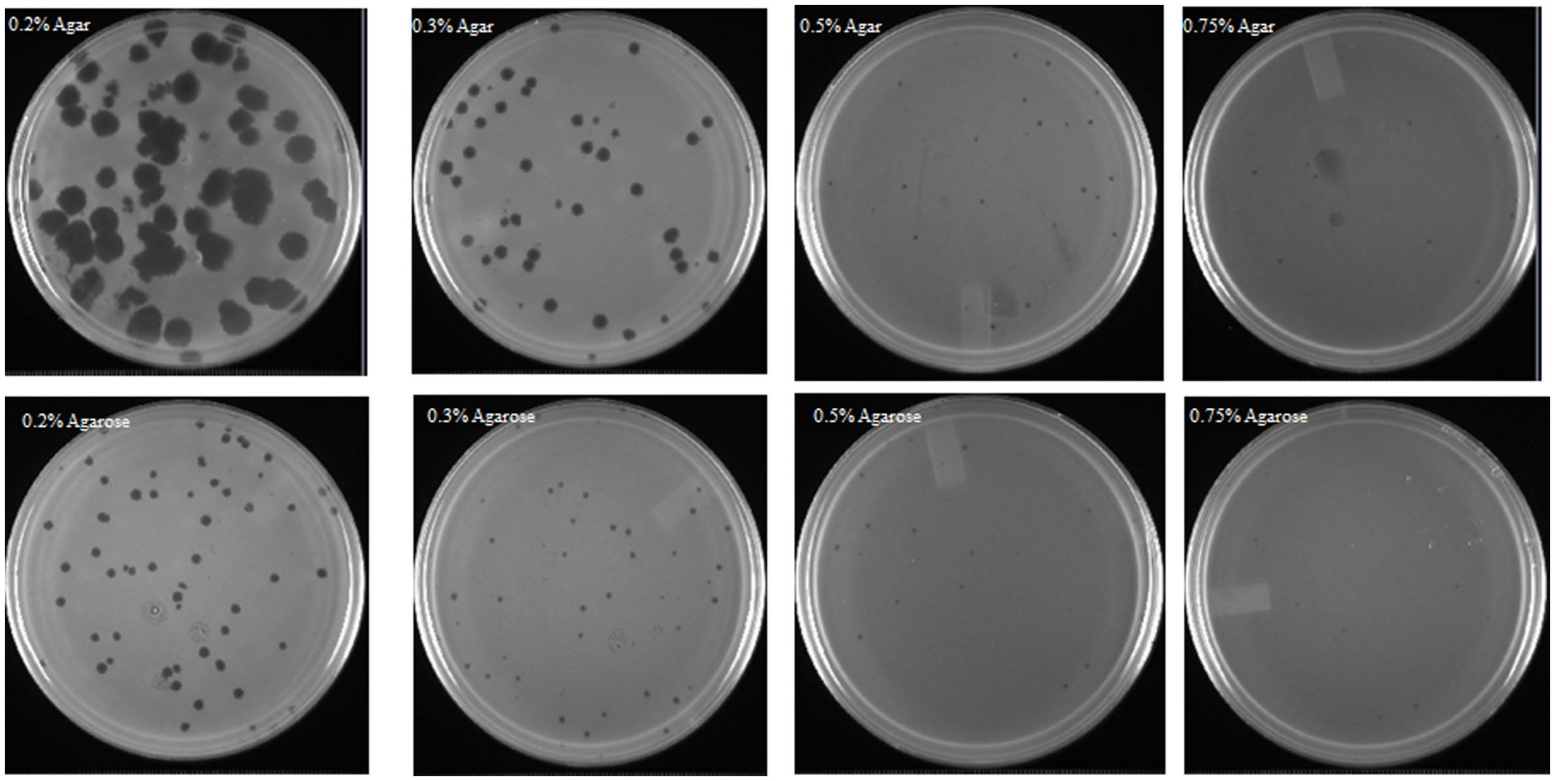
Influence of medium gelling agent (agar or agarose) and its concentration, in the soft overlay of the double-layer titer determination technique, on *Escherichia* bacteriophage OSYSP plaque formation and plaque size.

The conventional double-layer plaque assay (Kropinski et al., 2009) was modified to determine the effect of different gelling agents (agar or agarose) in the top layer (soft agar) on phage OSYSP titers. Decreasing the concentrations of these gelling agents from 0.7 to 0.2%, significantly (*p* < 0.05) increased the phage titers and the average diameter of the phage plaques (Figure 5). Enumerated phage titer was 0.7 log PFU/mL greater at 0.2% compared to 0.75% agar (Table 3). Moreover, phage plaque diameter dramatically (*p* < 0.05) increased from 0.08 cm to 0.74 cm with decreasing agar concentration from 0.75 to 0.2%. Similar changes in phage titers were observed when agar was replaced with agarose as the gelling agent; however, phage plaque diameters were significantly (*p* < 0.05) smaller when the agarose-incorporated top overlay was used for observations. Using 0.3% agar as a gelling agent in the top layer produced phage plaques with diameters that were double the size of those observed with 0.3% agarose overlay. There was no significant difference (*p* > 0.05) in phage titers when gelling agents (agar or agarose) were added at final concentrations of 0.2 or 0.3%; however, it took longer time for the top layer to harden when 0.2% of gelling agents were used. At 0.5 and 0.75% concentrations of the gelling agent in the top layer, the phage titers and plaque diameters sharply decreased compared to those observed at 0.2 and 0.3% concentrations. Therefore, 0.3% agar concentration was found optimum top layer gelling agent, for accurate and consistent phage titer determinations.

**Table 3:** Quantitative comparison of *Escherichia* bacteriophage OSYSP titers and plaque diameters at various agar or agarose concentrations used as a gelling agent in the soft overlay of the modified double-layer plaque assay^*^.

Concentrations (%)	0.20		0.30		0.50		0.75	
Gelling agent	Agar	Agarose	Agar	Agarose	Agar	Agarose	Agar	Agarose
Phage titers (log_10_ PFU/mL)	9.9 ± 0.01^a^	9.7 ± 0.02^b^	9.8 ± 0.06^a^	9.6 ± 0.05^b^	9.4 ± 0.13^c^	9.2 ± 0.11^c^	9.2 ± 0.06^d^	9.1 ± 0.07^e^
Phage plaque diameters (cm)	0.74 ± 0.09^a^	0.27 ± 0.03^b^	0.34 ± 0.03^c^	0.17 ± 0.02^d^	0.12 ± 0.02^e^	0.10 ± 0.03^e^	0.08 ± 0.02^ef^	0.07 ± 0.02^f^

* Each data point is the average from at least three independent trials. Error terms indicate ± standard deviations. Data points with different letter superscripts are statistically different at *p* < 0.05.

### 3.3 Phage OSYSP propagation dynamics

**Figure 6:**
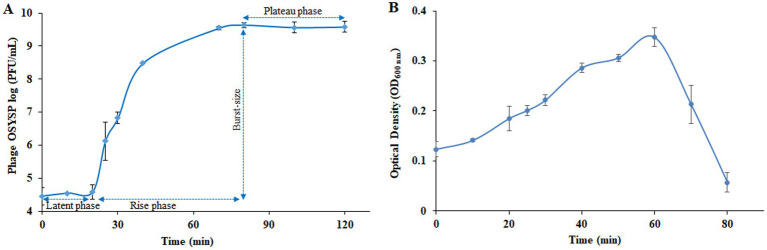
Changes in the populations of *Escherichia* bacteriophage OSYSP (phage OSYSP) and the host, *Escherichia coli* O157:H7 EDL933 during one-step growth curve experiments. **(A)** Phage OSYSP titers (log_10_ PFU/mL). **(B)** Turbidity changes, measured as OD_600nm_, due to changes in the population *E. coli* O157:H7 EDL933; corresponding population count changes are shown in Table 4. Each data point represents the average from three independent trials. Error bars indicate ± standard deviations.

Proliferation dynamics of phage OSYSP were determined from the one-step growth curve analysis (Figure 6A). The data showed that phage plaque count remained relatively stable over the first 20 min of growth. During this latent period, it is presumed that phage DNA was absorbed into the host cells, and viral elements were synthesized and assembled (Adams, 1959). Upon completion of assembly and lysing the cell, progeny phages were released from the host cell, resulting in a sharp increase in phage titer, as determined by plaque assay (Figure 6). Phage titer reached a plateau at the 80-min time point of phage multiplication curve, analogous to the stationary phase of a bacterial growth curve. Burst size was estimated by dividing the liberated phage titer by the initial population of infected host cells. Based on the one-step growth curve results, estimated burst size was 102 progeny phages per infected cell.

### 3.4 Phage OSYSP potency against *Escherichia coli* O157:H7 EDL933

Medium turbidity (OD_600 nm_) as a measure of bacterial host cell density, was monitored during 80-min phage propagation and results are shown in Figure 6B. The maximum OD_600 nm_ value (0.35) was observed at the 60-min of phage propagation, after which, OD decreased rapidly until reaching the lowest OD value (0.06) after 80 min of incubation, which coincides with peak phage titer. Based on these results, it can be concluded that phage OSYSP is highly lytic against the host *E. coli* O157:H7 EDL933, and it significantly (*p* < 0.05) decreased the cell population by more than 5.7 log CFU/mL during the 80-min incubation period (Table 4).

**Table 4:** *In vitro* potency of *Escherichia* bacteriophage OSYSP (Phage OSYSP) against the host, *Escherichia coli* O157:H7 EDL933^*^, measured during an 80-min one-step growth curve (Figure 6B).

Agent	Sampling time (min)	
	0	80
*E. coli* O157:H7 EDL933 (CFU/mL)	7.7 ± 0.07	<2.0^**^
Phage OSYSP (PFU/mL)	4.3 ± 0.7	9.7 ± 0.2

* Each data point represents the average from three independent trials. The error terms are ± standard deviations. Zero-time represents the time after the 20-min pre-infection incubation.

** The detection limit of the enumeration technique was 2.0 log CFU/mL.

### 3.5 Host range determination

**Figure 7:**
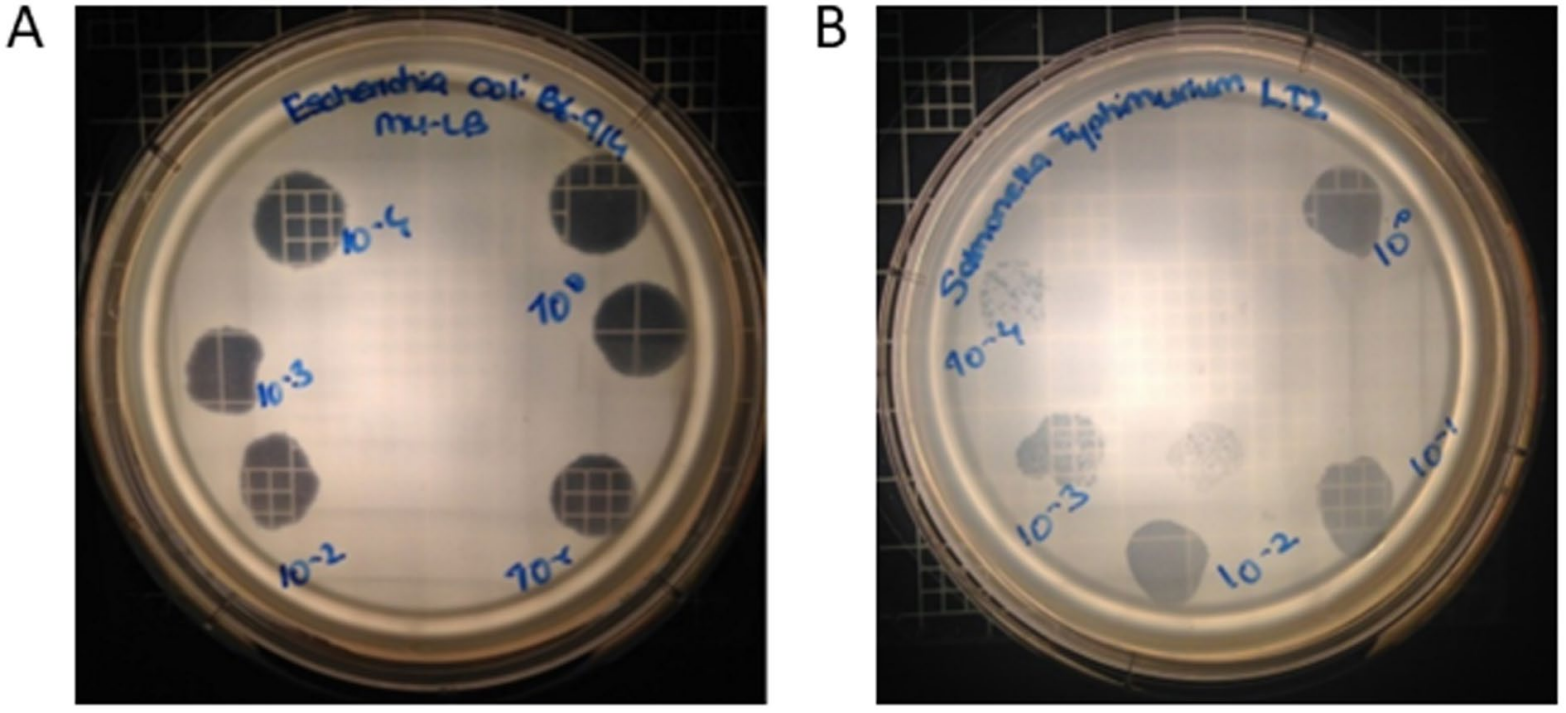
Effect of different *Escherichia* bacteriophage OSYSP titers on clear zone formation when spotting on a lawn of **(A)** *Escherichia coli* O157:H7 B6-914, and **(B)** *Salmonella* Typhimurium LT2. Concentration of *Escherichia* bacteriophage OSYSP stock suspension was 10^9^ PFU/mL; 10 μL of phage stock and its decimal dilutions were spotted, which corresponds to a dilution of 10^0^ (spotted on the upper right side of the plates) and subsequent dilutions, clock-wise, were 10^–1^ through 10^–4^.

Phage OSYSP lytic activity against host strain was measured by spot tests for ability to produce clear inhibition areas, and the phage’s ability to produce clear plaques from individual viral particles, i.e., phage titer. Five *E. coli* and 12 *S. enterica* strains were tested against phage OSYSP (Table 5). Among *E. coli* strains, the phage was highly effective against all O157:H7 strains, as observed from the clear inhibition zones and capability to produce phage titers. In addition to the pathogenic strains, phage OSYSP also infected and lysed *E. coli* K12, as determined by spot tests. Whole genome sequence of phage OSYSP revealed its potential broad-spectrum activity, as the phage shares genome similarities with phages previously found active against *Escherichia* and *Salmonella* strains (Hong et al., 2014; Park et al., 2012). Guided by this genomic information, we tested phage OSYSP against *Salmonella enterica* serovars. Results showed that serovars Typhimurium LT2, Javiana, Montevideo, and Tennessee were infected by the phage. Among these, *Salmonella* Tennessee produced both complete inhibition zones and phage plaques, however plaques were not clear (Supplementary Figure 1). Phage OSYSP produced clear inhibition zones when spotted on a lawn of *Salmonella* Typhimurium LT2, however, no phage plaques were observed. Diluting the phage stock from 10^9^ to 10^5^ PFU/mL before spot-testing on *E. coli* O157:H7 B6-914 or *Salmonella Typhimurium* LT2 seeded agar plates showed that the phage can infect and lyse both microorganisms (Figure 7); however, the activity was more pronounced against *E. coli* O157:H7 B6-914, even at the lower phage concentrations tested.

**Table 5:** Determination of *Escherichia* bacteriophage OSYSP host range using *Escherichia coli* strains and *Salmonella enterica* serovars (Supplementary Figure 1).

Strain	Inhibition area by spot test	Plaque formation during titer determination
*Escherichia coli* O157:H7 EDL933	+++	+++
*E. coli* O157:H7 B6-914	+++	+++
*E. coli* O157:H7 Sakai	+++	+++
*E. coli* K12	+++	+++
*E. coli* O157 E5	−	−
*Salmonella* Senftenberg	−	−
*Salmonella* Saintpaul	−	−
*Salmonella Enteritidis*	−	−
*Salmonella* Kentucky	−	−
*Salmonella Typhimurium* ATCC BAA-185	−	−
*Salmonella* Tennessee	+	++
*Salmonella* Poona	−	−
*Salmonella Typhimurium* LT2	+++	−
*Salmonella* Montevideo	++	−
*Salmonella* Javiana	++	−
*Salmonella* Eimsbuettel	−	−
*Salmonella Typhimurium* DT105	−	−

+++ Clear plaques or complete inhibition zones.

++ Clear plaques or complete inhibition zone with relatively low haziness.

+ Hazy plaques or inhibition zones with relatively high turbidity.

− Phage has no effect on host growth under the experimental conditions tested.

### 3.6 Phage OSYSP stability during 24-month cold storage

**Figure 8:**
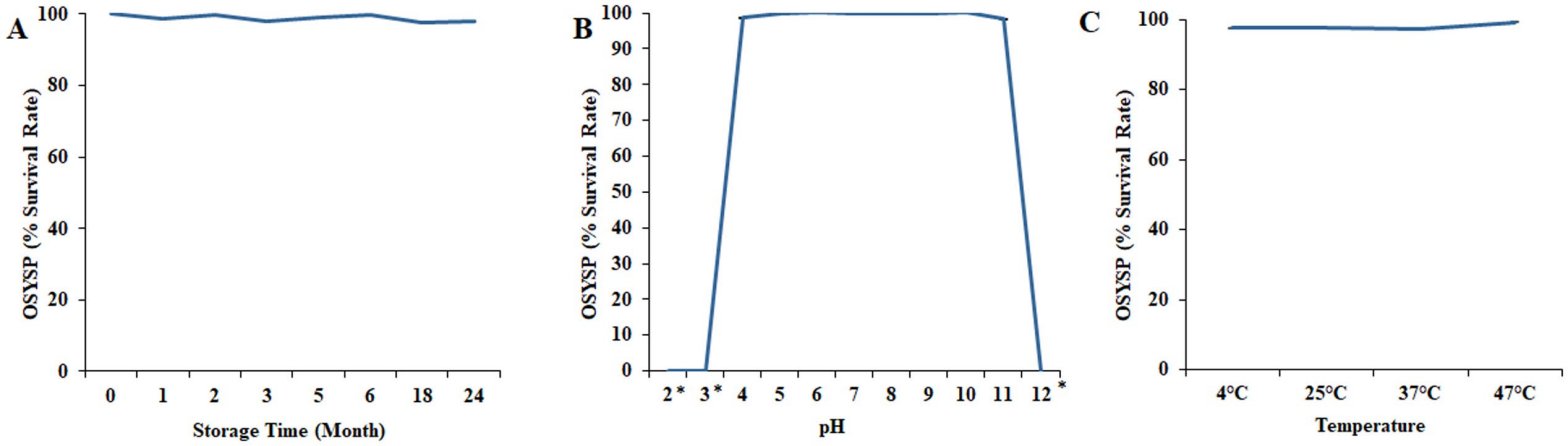
Changes in *Escherichia* bacteriophage OSYSP survival under nonoptimal conditions and longevity during lengthy storage. **(A)** Storage at 4°C for 24 months. **(B)** Incubation at different pH values for 30 min. **(C)** Incubation at different temperatures for 30 min. Each data point represents the average of three independent trials, except for the long-term storage, which was conducted once. Asterisks indicate the phage titer was lower than the detection limit (1 log PFU/mL) of the assay.

Long term shelf-life stability of phage OSYSP was evaluated by quantifying the phage at various time points during storage at 4°C (Figure 8A). Phage titers were relatively stable throughout the storage period, with the calculated phage count ranged between 9.1 and 9.4 log PFU/mL. The lytic activity was evaluated by measuring the average diameters of phage plaques in samples taken during the 24-month storage period. The results indicated no significant difference (*p* > 0.05) in the mean phage plaque diameters between fresh phage (0.31 ± 0.03 mm) and 2-year-old stock (0.32 ± 0.05 mm).

### 3.7 pH sensitivity

The influence of pH on phage OSYSP viability was investigated over the broad range of pH values, 2–12 (Figure 8B). Extreme pH values at 2, 3, and 12 adversely affected phage infectivity during 30-min treatment time at 37°C. At these pH levels, phage titers decreased dramatically (*p* < 0.05) from an average initial titer of 8.9 log PFU/mL to below the detection limit of the enumeration method (i.e., <1 log PFU/mL). However, at pH values ranging from 4 to 11, phage OSYSP titers remained fairly unchangeable (*p* > 0.05), compared to the average initial concentration of 8.9 log PFU/mL.

### 3.8 Stability at different incubation temperatures

The influence of four different incubation temperatures (4, 25, 37, 47°C) for 30 min on the viability of phage OSYSP in BPW (pH 7.3) is shown in Figure 8C. The average of initial phage titer was 9.7 log PFU/mL. Phage OSYSP was relatively stable across the temperatures studied, with no significant effect (*p >* 0.05) on phage infectivity. None of the temperature treatments changed the phage titers by more than a 0.2 log PFU/mL (*p* > 0.05).

## 4 Discussion

### 4.1 Genetic characterization

Estimating genome size is vital for understanding phage biodiversity. Phage OSYSP possesses a double-stranded DNA genome of 110,901 base pairs, comparable in size to closely related *Escherichia* phages: phiLLS (107,263 bp), vB_EcoS_FFH1 (108,483 bp), and vB_EcoS_AKFV33 (108,853 bp). In a previous study, the genome size of phage OSYSP was approximated at 150 kb using pulsed field gel electrophoresis (Snyder et al., 2016). However, the results of this study highlight the significance of whole genome sequencing for accurate phage genome size determination. Average nucleotide identity (ANIb) was used to compare phage OSYSP genome with relevant phage genomes. The genome of phage OSYSP showed a high similarity (93.11%) to phage phiLLS (KY677846.1), which has been reported to pack its DNA by using headful packaging strategy (Amarillas et al., 2017). In addition, phage OSYSP showed 93.10 and 92.55% similarities to T5-like phages FFH1 (KJ190157.1) and AKFV33 (HQ665011.1), respectively. However, phage OSYSP has a different genome organization compared to phages AKFV33 and FFH1, as confirmed by conventional PCR and additional whole genome data attained by Ion Torrent sequencing platform. It is widely recognized that comparative genomics of phages has unveiled the mosaic structure in their genomes. This pattern is probably a result of recombination events, wherein phages engage in horizontal gene transfer with other phages (Hatfull, 2008). Notably, the long exact direct repeat ends (12 kb) in typical T5 phages were not found in the phage OSYSP phage genome; suggesting that phage OSYSP may use a different DNA packaging strategy. Analyzing the amino acid sequence of *TerL* also revealed the close similarities among phage OSYSP, bacteriophage T5, and phage phiLLS; however, bacteriophage T5 phage and phiLLS use different DNA packaging strategies. Historically, similarity in G + C content percentage was considered valuable for virus classification (Ackermann, 2011). Both bacteriophage T5 and phage OSYSP have a GC content of 39.2%. Recently the International Committee on Taxonomy of Viruses (ICTV) abolished morphology-based families (Turner et al., 2021). Detailed analysis of the complete genome suggested that phage OSYSP is closely associated with T5-like lytic phages and belongs to the *Caudoviricetes* class.

For therapeutic or food safety purposes, a phage should exhibit a strictly lytic behavior. Genomic analysis of phage OSYSP confirms its obligate lytic life cycle. Phage OSYSP encodes genes for lytic enzymes and lacks the lysogenic determinants typically associated with temperate phages. Lytic phages utilize endolysins and holin proteins to successfully complete their reproductive life cycle. Phage OSYSP encodes two endolysins, also known as lysozymes, in its genome. Phage OSYSP endolysins have a close similarity with those from *Salmonella* phage Spc35 and *Escherichia* phage vB_EcoS_AKFV33, both of which were effective against their host bacteria (Kim and Ryu, 2011; Niu et al., 2012). Holin, a small membrane protein produced by phage, builds up in the host cell’s cytoplasm and triggers the destruction of bacterial cytoplasmic membrane. This allow endolysins to access and break down the peptidoglycan layer of host’s cell wall (Young, 2014). In the phage OSYSP genome, *Rz*-like spanins have also been identified. These proteins disrupt the outer membrane of the host cell during the release of progeny phages (Young, 2014). Moreover, *Rz*-like spanins, are closely related to those found in *Escherichia* and *Salmonella* phages, suggesting that phage OSYSP is well-equipped to efficiently lyse its host, making it a promising candidate for use in food safety and therapeutic applications.

Approximately 30% of the functional proteins in phage OSYSP genome are associated with tail and head proteins. Within the phage OSYSP genome, the tail length tape-measure protein has the longest amino acid sequence at 1227 amino acid residues, followed by the tail protein at 969 residues. The tail tape measure protein is crucial for determining tail length and facilitating the transfer of DNA into the cytoplasm of the host cell during infection (Mahony et al., 2016). Consistent with published literature, phage OSYSP genome encodes various tail proteins involved in receptor binding, phage DNA transmission, and tail sheath monomer function, all vital for recognizing and binding to bacterial hosts. Tail and head proteins of phage OSYSP showed high sequence similarity to potent *Salmonella* and *Escherichia* phages. The DNA packaging process in phage OSYSP involves genes encoded in orf 95, 97, and 98, which are responsible for the portal protein and both large and small terminase subunits. These proteins are known to be involved in DNA recognition, packaging phage DNA into an empty capsid, and its release from the capsid (Dedeo et al., 2019).

The DNA recombination, transcription, and repair system of phage OSYSP accounts for the majority of its functional proteins. These proteins are responsible for cleaving, recombining, and repairing DNA; this observation suggests that phage OSYSP has its own independent DNA recombination, transcription, and repair system. Phage OSYSP is a potential candidate for biocontrol agent because it does not carry any virulence genes, allergenic components, or antibiotic resistance related elements. Phage OSYSP also possesses 27 tRNAs, indicating that it is independent of host’s tRNA for protein synthesis. The absence of Cro, CI, CII, CIII, N, and Q proteins in the phage OSYSP genome indicates that it is not a temperate phage (Golding et al., 2019).

Phage propagation, purification, and DNA isolation steps require meticulous work before whole genome sequencing. Any host DNA contamination can adversely affect the genomic sequencing outcome. Some bacteriophages carry toxin genes that can confer these traits to their hosts (Herold et al., 2004). Thus, it is important to confirm the absence of toxin-producing genes in the phage genome for safe phage therapy. Although phage OSYSP genome was void of Shiga toxin genes, we observed these genes during a PCR experiment when phage OSYSP was propagated with the Shiga toxin-producing *E. coli* O157:H7 EDL933 (Figure 4). However, when the non-pathogenic surrogate, *E. coli* O157:H7 B6-914, was used in the propagation, PCR products did not reveal Shiga toxin genes. This confirms that the earlier toxin detections were likely due to DNA contaminants from the pathogenic host. Thus, phage OSYSP does not inherently contain these genes, making it a safe option for use in food safety and therapeutic applications.

### 4.2 Phage detection and quantification

For reliable phage research, it is vital to understand and maintain conditions that can ensure consistent phage detection and enumeration. Phage titer refers to the number of infective phages per mL of the liquid medium. Although different phage enumeration methods exist, the double-layer plague assay is the most common technique used (Kropinski et al., 2009). Several researchers have successfully modified this method by incorporating sublethal concentrations of antibiotics to trigger host cell lysis, when it is otherwise hard to detect and enumerate the phages plaques (Loś et al., 2008; Santos et al., 2009). Additionally, other researchers recommended using agarose at lower concentrations (0.15%), as a medium gelling agent, to observe long-genome phages (>200Kb), which remain viable but undetectable with traditional methods or at high (0.4%) agarose concentrations (Serwer et al., 2007). In this study, we tested the commonly used gelling agents, agar or agarose, which were incorporated into the soft overlay of the assay to observe the effect of type and concentration of these agents on phage enumeration. The type and concentration of the soft overlay gelling agent significantly affected phage titers and plaque diameters (Table 3). Using low agar concentrations (0.2 or 0.3%) enhanced both phage plaque diameters and phage titers. Agarose-incorporated overlays produced relatively smaller plaques compared to those using agar. In microbiological laboratories, agar and agarose remain transparent, whether hot or cooled, and do not adversely affect microbial growth (Armisén, 1991). Effective bacteriophage plaque formation relies on the successful movement of the phages to collide with susceptible host bacteria (Dennehy and Abedon, 2021). While agarose is a component of agar, it possesses greater gelling properties (Armisén, 1991). In the current study, using agarose instead of agar for the soft overlay restricted the phage’s ability to infect the host bacterium, an observation consistent with agarose superior gelling characteristics. This finding emphasizes the importance of using agar as the gelling agent, at relatively low concentrations, when enumerating phages to ensure accurate and consistent results.

### 4.3 Phage and host growth dynamics and interactions

Phage growth dynamics are commonly analyzed to predict the behavior of the phage against the target bacterium. Some of the growth parameters studied in the current study were latent period, burst size, and burst time. Depending on host strain density, cell size, and phage cultivation conditions used for the propagation of the phage, these parameters may show discrepancies (Wang et al., 1996). The 20-min latent period (time between the phage adsorption and initial release of the progeny phages) for phage OSYSP was found to be similar to those of phages FFH1 and SFP10, which have been considered potential biological control agents against *E. coli* O157:H7 (Hong et al., 2014; Park et al., 2012). Shorter latent periods were attributed to high density cell environment or incomplete utilization of host cell resources for progeny phage production (Abedon, 1989). Burst size is the number of progeny phage particles released by the lysis of a single infected bacterium (Delbrück, 1945). For phage OSYSP, the burst time was 80 min (Figure 6A) and estimated burst size was 102 PFU/infected cell. This burst size is lower than the 170 and 350 progeny phages reported for the closely related potential biocontrol agents, phiLLS (Amarillas et al., 2017) and AKFV33 (Niu et al., 2012), respectively, but higher than the 71 progeny phages reported for vB_EcoM_SQ17 (Zhou et al., 2022). Single-step growth curves depict the phage intracellular activities and replication cycles. The replication curve of phage OSYSP clearly demonstrated its obligatory lytic life cycle (Figure 6A).

Phage OSYSP fitness was confirmed *in vitro* by enumerating the growing host bacteria population during phage propagation (Figure 6B). Phage OSYSP significantly (*p* < 0.05) decreased the host *E. coli* O157:H7 EDL933 counts by more than 5.7 log CFU/mL, even at a MOI as low as 0.01 (Table 4). Lytic bacteriophages are capable of lysing bacterial cells through lysis-from-within or lysis-from-without pathways. The lysis-from-without pathway requires overwhelming numbers of phages on bacterial cells to inactivate them without progeny phage release (Abedon, 2011). The high lytic activity observed at 1:100 phage-to-infected cell ratio (MOI: 0.01) suggests that phage OSYSP could be effective against pathogens when used in moderate doses for both food safety and therapeutic purposes. This finding aligns with previous application of phage OSYSP on spinach leaves, which resulted in the inactivation of the *E. coli* O157:H7 strain B6-914 by 1.7–3.5 log CFU/g (Yesil et al., 2023); increased efficacy was observed as the phage-to-host ratio increased, highlighting the potential of phage OSYSP in phage therapy.

Bacteriophages are typically applied as a cocktail of different phages in controlling pathogens to overcome the challenges of narrow host range (Shokri et al., 2017) and potential bacterial resistance (Jassim and Limoges, 2014). Furthermore, due to their high specificity, bacteriophages do not alter the beneficial intestinal microbiota when applied therapeutically. Phage OSYSP has demonstrated activity against enterohemorrhagic *E. coli*, non-pathogenic *E. coli* K12, and certain *Salmonella* serovars. However, in a clinical setting, interactions with non-target bacteria could reduce phage OSYSP’s efficacy against the targeted pathogen. Further research is needed to confirm OSYSP’s selective therapeutic potential. The appearance of phage plaques and zones of clarity resulting from the phage’s lytic action suggests that phage OSYSP has an activity that was stronger against *E. coli* than *Salmonella*. Culture-based findings for host coverage are consistent with genome analysis, which showed that certain phage proteins, such as tail fiber proteins, have high sequence similarity with proteins found in both *Salmonella* and *Escherichia* phages. Previous mutagenesis studies have identified O antigens in both *Salmonella* and *E. coli* O157:H7 as receptors for phage SFP10 (Park et al., 2012). It is plausible that a similar host recognition mechanism exists in OSYSP; however, this hypothesis requires further experimental validation. Yet, phage OSYSP antibacterial activity was more evident in Shiga toxin-producing *E. coli*, even at the lower phage concentrations, demonstrating the potential efficacy of phage OSYSP as a single lytic-polyvalent-phage.

### 4.4 Phage longevity and survivability

In food safety and therapeutic applications, strictly lytic phages are ideal considering they can readily infect and lyse the host cells. To achieve such goals, the viability of these lytic phages must be preserved during short- or long-term storage before use. Some bacteriophages were found to be very sensitive to cold storage conditions (Van Tassel and Yousten, 1976). During 24-month cold storage, pure phage OSYSP maintained its activity without any adverse effect on its infectivity to the host. Additionally, environmental stressors (wide range of pH values and incubation temperatures) did not alter phage OSYSP infectivity. In therapeutic applications, it is likely that a single phage or phage cocktails would be administered through oral route and the phage would ideally survive the acidic gastrointestinal environment (Chibani-Chennoufi et al., 2004). In food applications, diminished phage activity was demonstrated in acidic foods (Oliveira et al., 2014). At extreme pH values of 2, 3 or 12, phage OSYSP lost its infectivity within 30 min; however, phage OSYSP survival rate was relatively unchanged at pH values between 4 and 11. Naturally acidic foods often inhibit the growth of many bacterial pathogens, whereas numerous processed foods are intentionally acidified and then heat treated to ensure microbiological safety and stability throughout their shelf-life (Watts, 2022; Dufort et al., 2017). Most foods, including meat and vegetables, typically have moderate pH values (Rahman, 2007), which fall within the effective pH range for phage OSYSP, making it a suitable candidate for food protection against *E. coli* O157:H7. Researchers also have demonstrated that potent bacteriophages can be encapsulated and safely employed in the acidic environment of gastrointestinal tract for therapeutic use (Yang et al., 2023). Temperature is another potential stressor for phages. Phage OSYSP remained stable during short incubation (30 min) at 4–47°C. While this duration might seem brief, it is sufficient for the phage to act against its host. Thermally stable phages have been shown to effectively control pathogens synergistically when used in combination with heat during egg decontamination (Yi et al., 2021). A recent publication on thermal stability of phage OSYSP (Yesil et al., 2024) highlighted its remarkable resilience at elevated temperatures relative to its host bacterium, and its potential use with other physical agents to improve microbial food safety while preserving the food quality. Given the resilience and stability of phage OSYSP under challenging environmental conditions, it emerges as a promising candidate for food or therapeutic applications.

## 5 Conclusion

Unlike synthetic preservatives, bacteriophages are biological control agents that rely on the host and environmental conditions to maintain their activity. Phage OSYSP was more effective against *E. coli* strains than against *Salmonella*, which signifies the importance of determining the permissive host to achieve the intended use of bacteriophages. Additionally, long term storage stability at refrigeration conditions is desirable for industrial use. This study highlights phage OSYSP’s robustness, evidenced by its 2-year stability in cold storage and its resilience in various environmental conditions, making it a viable candidate for industrial applications. By modifying the double-layer plaque assay, our results demonstrated that using low concentrations of agar in the soft overlay enhanced phage ability to infect the host bacterium and improved phage detection and enumeration technique. Molecular techniques helped assess the safety and suitability of phage OSYSP for food and medical applications. As a natural and effective biocontrol agent against foodborne Shiga toxin-producing *E. coli* and capable of withstanding adverse conditions, phage OSYSP is a promising biocontrol agent for food safety or therapeutic applications.

## Data Availability

Genome sequence of *Escherichia* phage OSYSP has been deposited at NCBI database with accession number MF402939.

## Footnotes

^1^ https://proksee.ca
