# Supplementary material for: Genomic analysis, culturing optimization, and characterization of Escherichia bacteriophage OSYSP, previously studied as effective pathogen control on fresh produce
Source: Front Microbiol. 2024 Dec 9;15:1486333. doi: 10.3389/fmicb.2024.1486333 (PMC11664485; doi:10.3389/fmicb.2024.1486333)
Supplement: Supplementary file 1 [file Presentation_1.pptx]

## Slide 1
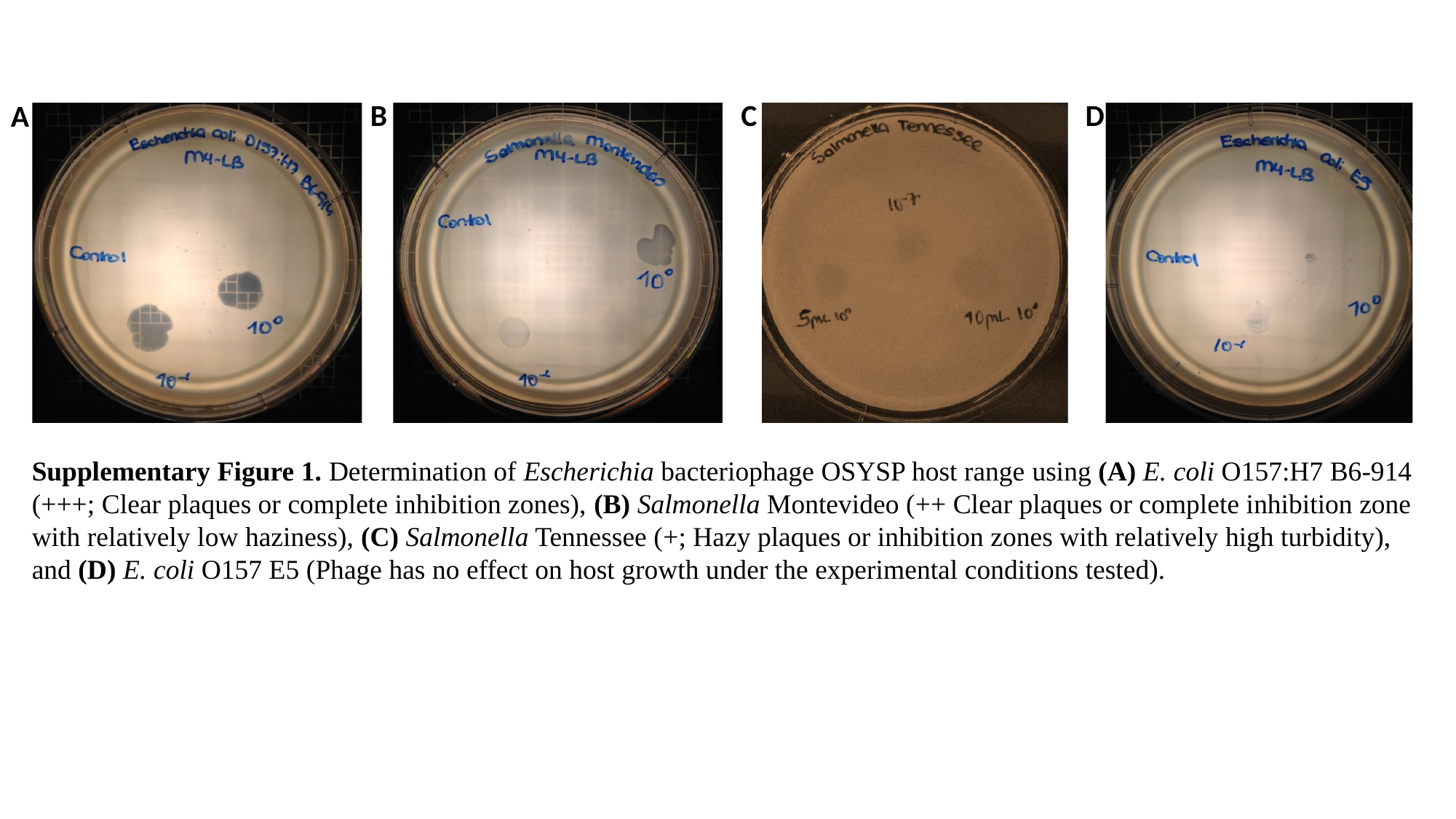

B
C
D
A
Supplementary Figure 1. Determination of Escherichia bacteriophage OSYSP host range using (A) E. coli O157:H7 B6-914 (+++; Clear plaques or complete inhibition zones), (B) Salmonella Montevideo (++ Clear plaques or complete inhibition zone with relatively low haziness), (C) Salmonella Tennessee (+; Hazy plaques or inhibition zones with relatively high turbidity), and (D) E. coli O157 E5 (Phage has no effect on host growth under the experimental conditions tested).
