# Supplementary material for: Genomic analysis, culturing optimization, and characterization of Escherichia bacteriophage OSYSP, previously studied as effective pathogen control on fresh produce
Source: Front Microbiol. 2024 Dec 9;15:1486333. doi: 10.3389/fmicb.2024.1486333 (PMC11664485; doi:10.3389/fmicb.2024.1486333)
Supplement: Supplementary file 2 [file Table_1.docx]

**Supplementary Table 1.** Gene products of *Escherichia* phage OSYSP genome

|  | | | | | |
| --- | --- | --- | --- | --- | --- |
| **Number** | **NCBI Protein ID** | **Start** | **End** | **Strand** | **Protein Name** |
| 1 | YP_009790924.1 | 160 | 528 | - | Ribonucleotide reductase subunit |
| 2 | YP_009790925.1 | 614 | 802 | - | Hypothetical protein |
| 3 | YP_009790926.1 | 1058 | 1351 | - | Hypothetical protein |
| 4 | YP_009790927.1 | 1511 | 1675 | - | Hypothetical protein |
| 5 | YP_009790928.1 | 1668 | 1889 | - | Hypothetical protein |
| 6 | YP_009790929.1 | 2157 | 2351 | - | Hypothetical protein |
| 7 | YP_009790930.1 | 2366 | 2497 | - | Hypothetical protein |
| 8 | YP_009790931.1 | 2821 | 3003 | - | Hypothetical protein |
| 9 | YP_009790932.1 | 3123 | 3470 | - | Hypothetical protein |
| 10 | YP_009790933.1 | 4114 | 4467 | - | Hypothetical protein |
| 11 | YP_009790934.1 | 4557 | 4700 | - | Hypothetical protein |
| 12 | YP_009790935.1 | 4700 | 5017 | - | Hypothetical protein |
| 13 | YP_009790936.1 | 5115 | 5282 | - | Hypothetical protein |
| 14 | YP_009790937.1 | 5469 | 5627 | - | Hypothetical protein |
| 15 | YP_009790938.1 | 6432 | 6779 | - | Hypothetical protein |
| 16 | YP_009790939.1 | 6892 | 7077 | - | Hypothetical protein |
| 17 | YP_009790940.1 | 7332 | 7523 | - | Hypothetical protein |
| 18 | YP_009790941.1 | 7524 | 7742 | - | Hypothetical protein |
| 19 | YP_009790942.1 | 7793 | 8065 | - | Hypothetical protein |
| 20 | YP_009790943.1 | 8543 | 8818 | - | Hypothetical protein |
| 21 | YP_009790944.1 | 8909 | 9115 | - | Hypothetical protein |
| 22 | YP_009790945.1 | 9108 | 9224 | - | Hypothetical protein |
| 23 | YP_009790946.1 | 9475 | 9654 | - | Anti-termination protein Q-like |
| 24 | YP_009790947.1 | 10068 | 10586 | - | DNA primase |
| 25 | YP_009790948.1 | 10853 | 11833 | + | Hypothetical protein |
| 26 | YP_009790949.1 | 11855 | 12271 | + | Hypothetical protein |
| 27 | YP_009790950.1 | 12596 | 13543 | - | Lipoprotein |
| 28 | YP_009790951.1 | 14487 | 14930 | - | tRNA amidotransferase |
| 29 | YP_009790952.1 | 14930 | 15100 | - | Hypothetical protein |
| 30 | YP_009790953.1 | 15169 | 15618 | - | Endolysin |
| 31 | YP_009790954.1 | 15624 | 15941 | - | Hypothetical protein |
| 32 | YP_009790955.1 | 16384 | 17022 | - | Tail fiber protein |
| 33 | YP_009790956.1 | 17076 | 17258 | - | Hypothetical protein |
| 34 | YP_009790957.1 | 17329 | 18030 | - | Exonuclease |
| 35 | YP_009790958.1 | 18059 | 18295 | - | Hypothetical protein |
| 36 | YP_009790959.1 | 18337 | 18552 | - | Baseplate wedge subunit |
| 37 | YP_009790960.1 | 18617 | 19132 | - | Virion structural protein |
| 38 | YP_009790961.1 | 19208 | 19657 | - | Swarming motility protein |
| 39 | YP_009790962.1 | 19654 | 19938 | - | Hypothetical protein |
| 40 | YP_009790963.1 | 20015 | 20491 | - | Ribonuclease H |
| 41 | YP_009790964.1 | 20491 | 20691 | - | Hypothetical protein |
| 42 | YP_009790965.1 | 20786 | 21625 | - | Thymidylate synthase |
| 43 | YP_009790966.1 | 21625 | 22158 | - | Putative dihydrofolate reductase |
| 44 | YP_009790967.1 | 22155 | 23300 | - | Aerobic ribonucleoside diphosphate reductase, small subunit |
| 45 | YP_009790968.1 | 23408 | 25738 | - | Ribonucleoside diphosphate reductase, large subunit |
| 46 | YP_009790969.1 | 25860 | 26102 | - | Tail length tape measure protein |
| 47 | YP_009790970.1 | 26104 | 26856 | - | Phosphate starvation-inducible protein |
| 48 | YP_009790971.1 | 27227 | 29101 | + | Anaerobic ribonucleoside-triphosphate reductase |
| 49 | YP_009790972.1 | 29201 | 29482 | + | Hypothetical protein |
| 50 | YP_009790973.1 | 29492 | 29698 | + | Hypothetical protein |
| 51 | YP_009790974.1 | 29682 | 29885 | + | Hypothetical protein |
| 52 | YP_009790975.1 | 29878 | 30702 | + | Sir2 (NAD-dependent deacetylase) |
| 53 | YP_009790976.1 | 30693 | 30875 | + | Hypothetical protein |
| 54 | YP_009790977.1 | 30862 | 31368 | + | Sir2 (NAD-dependent deacetylase) |
| 55 | YP_009790978.1 | 31371 | 31799 | + | Hypothetical protein |
| 56 | YP_009790979.1 | 31809 | 32207 | + | Hypothetical protein |
| 57 | YP_009790980.1 | 32962 | 35616 | + | Replication origin binding protein |
| 58 | YP_009790981.1 | 35600 | 35833 | + | Hypothetical protein |
| 59 | YP_009790982.1 | 35903 | 36607 | + | Helicase |
| 60 | YP_009790983.1 | 36955 | 37365 | + | DNA binding protein |
| 61 | YP_009790984.1 | 37402 | 37698 | + | Hypothetical protein |
| 62 | YP_009790985.1 | 37749 | 38057 | + | Transcriptional regulator |
| 63 | YP_009790986.1 | 38145 | 39116 | + | NAD-dependent DNA ligase, subunit A |
| 64 | YP_009790987.1 | 39319 | 40095 | + | NAD-dependent DNA ligase subunit B |
| 65 | YP_009790988.1 | 40088 | 40855 | + | DNA-binding protein |
| 66 | YP_009790989.1 | 40938 | 42410 | + | Putative replicative DNA helicase |
| 67 | YP_009790990.1 | 42407 | 43297 | + | DNA primase |
| 68 | YP_009790991.1 | 43360 | 45927 | + | DNA polymerase |
| 69 | YP_009790992.1 | 46130 | 46417 | + | Hypothetical protein |
| 70 | YP_009790993.1 | 46414 | 47766 | + | putative ATP-dependent helicase |
| 71 | YP_009790994.1 | 47989 | 48273 | + | Hypothetical protein |
| 72 | YP_009790995.1 | 48266 | 49039 | + | Single strand DNA binding protein |
| 73 | YP_009790996.1 | 49076 | 50053 | + | Calcineurin-like phosphoesterase superfamily domain protein |
| 74 | YP_009790997.1 | 50034 | 51872 | + | Putative exonuclease SbcCD C subunit |
| 75 | YP_009790998.1 | 51876 | 52358 | + | RusA-like Holliday junction resolvase |
| 76 | YP_009790999.1 | 52358 | 53233 | + | Flap endonuclease |
| 77 | YP_009791000.1 | 53230 | 53676 | + | dUTPase |
| 78 | YP_009791001.1 | 53654 | 53896 | + | Hypothetical protein |
| 79 | YP_009791002.1 | 53954 | 56863 | - | Tail fiber protein |
| 80 | YP_009791003.1 | 56863 | 57285 | - | Collar tail protein for L-shaped tail fibre attachment |
| 81 | YP_009791004.1 | 57292 | 59349 | - | Straight fibre tail protein |
| 82 | YP_009791005.1 | 59349 | 62198 | - | Tail protein |
| 83 | YP_009791006.1 | 62195 | 62809 | - | Distal tail protein |
| 84 | YP_009791007.1 | 62919 | 66602 | - | Tail length tape-measure protein |
| 85 | YP_009791008.1 | 66686 | 67018 | - | Tail assembly chaperone |
| 86 | YP_009791009.1 | 67116 | 67520 | - | Tail assembly chaperone |
| 87 | YP_009791010.1 | 67517 | 68416 | - | Base plate tail tube protein |
| 88 | YP_009791011.1 | 68421 | 69818 | - | Major tail protein |
| 89 | YP_009791012.1 | 69845 | 70330 | - | Tail terminator protein |
| 90 | YP_009791013.1 | 70334 | 71101 | - | Tail completion or Neck1protein |
| 91 | YP_009791014.1 | 71101 | 71613 | - | Head-tail adaptor |
| 92 | YP_009791015.1 | 71672 | 73048 | - | Major head protein |
| 93 | YP_009791016.1 | 73066 | 73698 | - | Head maturation protease |
| 94 | YP_009791017.1 | 73702 | 74196 | - | Hoc-like head decoration |
| 95 | YP_009791018.1 | 74193 | 75410 | - | Portal protein |
| 96 | YP_009791019.1 | 75410 | 75847 | - | Hypothetical protein |
| 97 | YP_009791020.1 | 75962 | 77278 | - | Terminase large subunit |
| 98 | YP_009791021.1 | 77278 | 77760 | - | Terminase small subunit |
| 99 | YP_009791022.1 | 77771 | 79558 | - | Receptor binding tail protein |
| 100 | YP_009791023.1 | 79644 | 79910 | + | Cor superinfection exclusion protein |
| 101 | YP_009791024.1 | 80024 | 80215 | + | Hypothetical protein |
| 102 | YP_009791025.1 | 80215 | 80322 | + | Hypothetical protein |
| 103 | YP_009791026.1 | 80319 | 80513 | + | Tail assembly protein |
| 104 | YP_009791027.1 | 80506 | 80751 | + | Membrane protein |
| 105 | YP_009791028.1 | 80904 | 81638 | - | Deoxynucleoside-5'-monophosphatase |
| 106 | YP_009791029.1 | 81719 | 82111 | - | Hypothetical protein |
| 107 | YP_009791030.1 | 82150 | 82407 | - | Hypothetical protein |
| 108 | YP_009791031.1 | 82463 | 84127 | - | DNA transfer protein |
| 109 | YP_009791032.1 | 84224 | 84421 | - | Membrane protein |
| 110 | YP_009791033.1 | 84478 | 84885 | - | DNA-binding protein |
| 111 | YP_009791034.1 | 84984 | 85235 | - | Baseplate wedge protein |
| 112 | YP_009791035.1 | 85463 | 85666 | - | Hypothetical protein |
| 113 | YP_009791036.1 | 86816 | 87808 | + | Hypothetical protein |
| 114 | YP_009791037.1 | 87873 | 88367 | + | Hypothetical protein |
| 115 | YP_009791038.1 | 88473 | 88703 | + | Hypothetical protein |
| 116 | YP_009791039.1 | 88750 | 88899 | + | Hypothetical protein |
| 117 | YP_009791040.1 | 88902 | 89114 | + | Endonuclease nicking abasic sites generated by host uracil glycosylase Ung |
| 118 | YP_009791041.1 | 89238 | 89582 | + | Hypothetical protein |
| 119 | YP_009791042.1 | 89579 | 89737 | + | Hypothetical protein |
| 120 | YP_009791043.1 | 89757 | 89951 | + | Hypothetical protein |
| 121 | YP_009791044.1 | 90852 | 90935 | + | Hypothetical protein |
| 122 | YP_009791045.1 | 91663 | 92019 | - | Homing endonuclease |
| 123 | YP_009791046.1 | 92006 | 92737 | - | Hypothetical protein |
| 124 | YP_009791047.1 | 92737 | 93357 | - | Hypothetical protein |
| 125 | YP_009791048.1 | 93357 | 93542 | - | Hypothetical protein |
| 126 | YP_009791049.1 | 93542 | 93775 | - | Hypothetical protein |
| 127 | YP_009791050.1 | 94046 | 94492 | - | Hypothetical protein |
| 128 | YP_009791051.1 | 94494 | 94697 | - | Hypothetical protein |
| 129 | YP_009791052.1 | 94754 | 95125 | - | Hypothetical protein |
| 130 | YP_009791053.1 | 95073 | 95534 | - | Hypothetical protein |
| 131 | YP_009791054.1 | 95531 | 95731 | - | Hypothetical protein |
| 132 | YP_009791055.1 | 95831 | 96157 | - | Hypothetical protein |
| 133 | YP_009791056.1 | 96147 | 96392 | - | Hypothetical protein |
| 134 | YP_009791057.1 | 96389 | 96670 | - | Tail sheath monomer |
| 135 | YP_009791058.1 | 96670 | 96834 | - | Hypothetical protein |
| 136 | YP_009791059.1 | 96834 | 97085 | - | Hypothetical protein |
| 137 | YP_009791060.1 | 97164 | 97595 | - | Hypothetical protein |
| 138 | YP_009791061.1 | 97648 | 97770 | - | Hypothetical protein |
| 139 | YP_009791062.1 | 97763 | 98353 | - | Phosphoesterase |
| 140 | YP_009791063.1 | 98353 | 98640 | - | Hypothetical protein |
| 141 | YP_009791064.1 | 98640 | 99503 | - | NinI-like serine-threonine phosphatase |
| 142 | YP_009791065.1 | 99506 | 99751 | - | Major head protein |
| 143 | YP_009791066.1 | 99849 | 100139 | - | Putative thioredoxin |
| 144 | YP_009791067.1 | 100132 | 100563 | - | Hypothetical protein |
| 145 | YP_009791068.1 | 100639 | 101055 | - | Hypothetical protein |
| 146 | YP_009791069.1 | 101133 | 101546 | - | Endolysin |
| 147 | YP_009791070.1 | 101543 | 102199 | - | Holin |
| 148 | YP_009791071.1 | 102457 | 102957 | - | ATP-dependent protease |
| 149 | YP_009791072.1 | 102970 | 103722 | - | Deoxynucleoside monophosphate kinase |
| 150 | YP_009791073.1 | 103722 | 104075 | - | Rz-like spanin |
| 151 | YP_009791074.1 | 104006 | 104449 | - | Rz-like spanin |
| 152 | YP_009791075.1 | 104449 | 104946 | - | H-N-H-endonuclease F-TflVI |
| 153 | YP_009791076.1 | 104943 | 105641 | - | Hypothetical protein |
| 154 | YP_009791077.1 | 105796 | 106140 | - | Hypothetical protein |
| 155 | YP_009791078.1 | 106251 | 106535 | - | Hypothetical protein |
| 156 | YP_009791079.1 | 106782 | 107201 | - | Hypothetical protein |
| 157 | YP_009791080.1 | 107194 | 107493 | - | Hypothetical protein |
| 158 | YP_009791081.1 | 107486 | 107767 | - | Hypothetical protein |
| 159 | YP_009791082.1 | 107844 | 108191 | - | Hypothetical protein |
| 160 | YP_009791083.1 | 108709 | 109077 | - | Acetyltransferase |
| 161 | YP_009791084.1 | 109127 | 109321 | - | Hypothetical protein |
| 162 | YP_009791085.1 | 109429 | 109512 | - | Hypothetical protein |
| 163 | YP_009791086.1 | 109609 | 109815 | - | Hypothetical protein |
| 164 | YP_009791087.1 | 109815 | 110108 | - | Hypothetical protein |
| 165 | YP_009791088.1 | 110268 | 110432 | - | Hypothetical protein |
| 166 | YP_009791089.1 | 110425 | 110676 | - | Hypothetical protein |
