# Supplementary material for: Genomic analysis, culturing optimization, and characterization of Escherichia bacteriophage OSYSP, previously studied as effective pathogen control on fresh produce
Source: Front Microbiol. 2024 Dec 9;15:1486333. doi: 10.3389/fmicb.2024.1486333 (PMC11664485; doi:10.3389/fmicb.2024.1486333)
Supplement: Supplementary file 3 [file Table_2.docx]

| **Supplementary Table 2.** The tRNA coding regions in phage OSYSP genome | | | | |
| --- | --- | --- | --- | --- |
| **tRNA** | **Begin** | **End** | **Amino acid** | **Anticodon** |
| 1 | 899 | 974 | Met | CAT |
| 2 | 981 | 1056 | Ile | GAT |
| 3 | 1426 | 1500 | Thr | TGT |
| 4 | 1905 | 1979 | Gly | TCC |
| 5 | 1986 | 2061 | Gln | TTG |
| 6 | 2068 | 2143 | Gln | CTG |
| 7 | 2377 | 2453 | His | GTG |
| 8 | 3020 | 3109 | Ser | TGA |
| 9 | 3693 | 3769 | Leu | TAG |
| 10 | 3775 | 3853 | Ala | TGC |
| 11 | 4493 | 4566 | Val | TAC |
| 12 | 5035 | 5113 | Lys | TTT |
| 13 | 5303 | 5380 | Met | CAT |
| 14 | 5387 | 5464 | Pro | TGG |
| 15 | 6806 | 6882 | Asp | GTC |
| 16 | 7180 | 7262 | Asn | GTT |
| 17 | 7270 | 7345 | Cys | GCA |
| 18 | 8083 | 8157 | Phe | GAA |
| 19 | 8244 | 8318 | Glu | TTC |
| 20 | 8327 | 8414 | Tyr | GTA |
| 21 | 9291 | 9367 | Leu | TAA |
| 22 | 9958 | 10046 | Ser | GCT |
| 23 | 13581 | 13652 | Arg | TCT |
| 24 | 109359 | 109434 | Met | CAT |
| 25 | 109531 | 109637 | Ile | GAT |
| 26 | 110183 | 110257 | Thr | TGT |
| 27 | 110776 | 110851 | Gln | TTG |
